# Natural and induced variations in transcriptional regulator genes result in low‐nicotine phenotypes in tobacco

**DOI:** 10.1111/tpj.15923

**Published:** 2022-08-11

**Authors:** Tsubasa Shoji, Koki Moriyama, Nicolas Sierro, Sonia Ouadi, Nikolai V. Ivanov, Takashi Hashimoto, Kazuki Saito

**Affiliations:** ^1^ RIKEN Center for Sustainable Resource Science, Tsurumi‐ku Yokohama Kanagawa 230‐0045 Japan; ^2^ Division of Biological Science Nara Institute of Science and Technology Ikoma Nara 630‐0101 Japan; ^3^ PMI R&D, Philip Morris Products S.A. Quai Jeanrenaud 5 CH‐2000 Neuchâtel Switzerland; ^4^ Plant Molecular Science Center Chiba University, Chuo‐ku Chiba 260‐8675 Japan

**Keywords:** alkaloids, ethylene response factor, low‐nicotine phenotype, nicotine, tobacco, transcription factor

## Abstract

In tobacco, the homologous ETHYLENE RESPONSE FACTOR (ERF) transcription factors ERF199 and ERF189 coordinate the transcription of multiple metabolic genes involved in nicotine biosynthesis. Natural alleles at the *NIC1* and *NIC2* loci greatly affect alkaloid accumulation and overlap with *ERF199* and *ERF189* in the tobacco genome, respectively. In this study, we identified several low‐nicotine tobacco varieties lacking *ERF199* or *ERF189* from a tobacco germplasm collection. We characterized the sequence of these new *nic1* and *nic2* alleles, as well as the previously defined alleles *nic1‐1* and *nic2‐1*. Moreover, we examined the influence of different *nic* alleles on alkaloid contents and expression levels of genes related to nicotine biosynthesis. We also demonstrated that the deletion of a distal genomic region attenuates *ERF199* expression, resulting in a moderately negative effect on the alkaloid phenotype. Our study provides new insights into the regulation of nicotine biosynthesis and novel genetic resources to breed low‐nicotine tobacco.

## Significance Statement

Reducing the accumulation of addictive substance nicotine is a major focus of tobacco breeding to mitigate health concerns. We studied the genomic structures and phenotypic effects of an allelic series for the *NIC1* and *NIC2* genes, which encode ETHYLENE RESPONSE FACTOR transcription factors that regulate nicotine biosynthesis, revealing the genomic basis of low‐nicotine phenotypes in tobacco.

## INTRODUCTION

Plants have tremendous metabolic plasticity to synthesize diverse natural products (Wurtzel & Kutchan, 2016). Genetic studies largely relying on a wide range of mutations or variants have greatly facilitated the molecular identification of metabolic enzymes and regulatory factors underlying metabolic processes in plants. A hallmark example is the molecular elucidation of the flavonoid biosynthetic pathways that produce the anthocyanin pigments (Cappellini et al., 2021). However, genetic variants associated with the accumulation of colorless natural products, including bioactive alkaloids and terpenoids, are more difficult to collect and characterize than those related to pigments are (Hibi et al., 1994; Millgate et al., 2004; Qin et al., 2010). Therefore, such studies lagged behind until recent advances in genomics and metabolomics (Shoji et al., 2021).

Tobacco (*Nicotiana tabacum*) roots produce pyridine alkaloids, such as nicotine, nornicotine, anabasine, and anatabine (Figure S1). These compounds then translocate through the xylem and accumulate mainly in the leaves as defense compounds against insect predators (Dewey & Xie, 2013; Shoji, 2020). Tobacco alkaloids are derived from amino acid precursors transformed through branched pathways, which depend on a number of metabolic enzymes, including putrescine *N*‐methyltransferase (PMT), quinolinate phosphoribosyl transferase 2 (QPT2), and the PIP‐family oxidoreductase A622 (Figure S1) (Dewey & Xie, 2013; Hibi et al., 1994; Kajikawa et al., 2009; Shoji, 2020; Shoji & Hashimoto, 2011). In the nicotine biosynthetic pathway, the expression of multiple metabolism and transport genes is coordinately induced by a homologous pair of ETHYLENE RESPONSE FACTOR (ERF) transcription factors, ERF199 and ERF189, which specifically recognize GC‐rich elements present in the promoter regions of their downstream genes (Shoji et al., 2010; Shoji et al., 2013; Shoji & Yuan, 2021).

Nicotine is the predominant alkaloid in tobacco and the main addictive substance in tobacco products. Therefore, lowering nicotine content has become a major focus of tobacco breeding to mitigate health concerns (WHO, 2015). Naturally, occurring genetic variants with low nicotine accumulation have become increasingly important in this context. *NIC1* and *NIC2* loci have a large influence on alkaloid accumulation in tobacco. These loci were identified through genetic analysis of a low‐nicotine phenotype originating from the natural variant of Cuban cigar tobacco (Valleau, 1949). The introgression of the low‐nicotine trait into the burley tobacco variety Burley21 and flue‐cure tobacco variety NC95 generated a series of near‐isogenic lines with different alkaloid contents (Hibi et al., 1994; Legg & Collins, 1971; Valleau, 1949; Legg et al., 1970; Chaplin, 1975). Their accumulation levels decrease in the following order: wild‐type, *nic2‐1*, *nic1‐1*, and *nic1‐1 nic2‐1* genotypes (*nic1‐1* and *nic2‐1* are used here to designate the two natural alleles in *NIC* and *NIC2*, respectively).

The *NIC2* locus is located on chromosome 19 and comprises several genes encoding ERF transcription factors, including *ERF189* (Kajikawa et al., 2017; Shoji et al., 2010). Clustered *ERF*s were found to be deleted in the *nic2‐1* mutant (Kajikawa et al., 2017, Shoji et al., 2010). The *NIC1* locus is located on chromosome 7, in a region containing multiple *ERF* genes related to those present in the *NIC2* locus, including *ERF199*, a homolog of *ERF189* (Qin et al., 2021; Sui et al., 2020). In the *nic1‐1* mutant, *ERF199* expression is lower than in the wild type, and the genomic regions downstream of *ERF199* are deleted (Qin et al., 2021, Sui et al., 2020). The mechanistic basis of the mutation has remained elusive; it is thus unknown why *nic1‐1* has a stronger effect on alkaloid phenotypes than *nic2‐1* (Hibi et al., 1994; Legg & Collins, 1971). In addition to the natural alleles *nic1‐1* and *nic2‐1*, complete loss‐of‐function alleles of *ERF199* and *ERF189*, named *nic1‐2* and *nic2‐2* respectively, were generated through CRISPR/Cas9‐mediated editing, and low‐nicotine phenotype was demonstrated in the *nic1‐2 nic2‐2* double mutant (Hayashi et al., 2020).

In this study, we selected tobacco accessions with a low‐nicotine phenotype from a germplasm collection (Sisson & Saunders, 1982). We determined the genomic sequences of the newly obtained *nic* alleles and the previously identified *nic1‐1* and *nic2‐1* alleles. In addition, we examined the influence conferred by distinct alleles on alkaloid contents and associated gene expression to gain insights into the molecular basis of low‐alkaloid phenotypes in tobacco. After generating a new *nic* allele by CRISPR/Cas9‐mediated editing, we demonstrated that the deletion of a one genomic region downstream of *ERF199* attenuates its expression in the *nic1* mutant alleles and had a moderate but significant effect on the alkaloid phenotype.

## RESULTS

### Genomic sequences of *nic1‐1* and *nic2‐1* alleles

To clarify the exact genomic structures at the *NIC1* and *NIC2* loci, we resequenced the genomes of the wild type, *nic1‐1* (registered as LI Burley 21), and *nic1‐1 nic2‐1* (registered as LA Burley 21) (Legg et al., 1970) in the Burley 21 background, as well as those of the wild type and *nic1‐1 nic2‐1* (registered as LAFC53) (Chaplin, 1975) in the NC95 background. We then mapped the sequencing reads to a chromosome‐anchored reference genome sequence for the tobacco variety K326 (manuscript in preparation).

**Figure 1:**
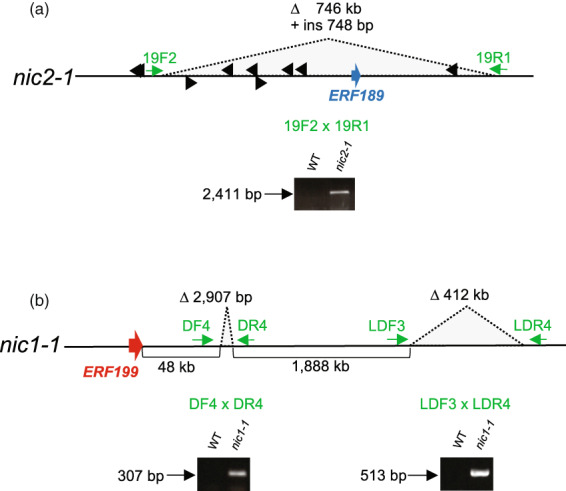
Genomic deletions in *nic1‐1* and *nic2‐1* alleles. Schematic diagrams of the deletions in the *nic2‐1* (a) and *nic1‐1* (b) alleles. Green arrows indicate the primers used for polymerase chain reaction (PCR) analysis, the red arrow indicates *ERF199*, the blue arrow indicates *ERF189*, and black arrowheads indicate other *ERF*s clustered in the *NIC2* locus. The deletions were detected by the PCR amplification of genomic fragments (2411‐bp fragment for *nic2‐1* and 307‐ and 513‐bp fragments for *nic1‐1*) with the indicated primers, while the wild‐type (WT) intact allele cannot be amplified. WT, *nic1‐1*, and *nic2‐1* genotypes in the Burley21 background were analyzed.

We had detected a large chromosomal deletion (approximately >650 kb) encompassing *ERF189* and its homologs in *nic2‐1* using genomic polymerase chain reaction (PCR) and DNA blot analyses (Kajikawa et al., 2017; Shoji et al., 2010). Genome resequencing delimited a possible deletion region between 142 775 000 and 143 521 250 bp on chromosome 19. We designed primers annealing to either side of the deletion to amplify over this region in *nic2‐1*. PCR amplifications with primers 19F2 and 19R1 yielded a clear PCR amplicon of 2411 bp (Figure 1a). Sequencing of the amplified fragment revealed a deletion of approximately 746 kb between 142 774 939 and 143 521 207 bp on chromosome 19, together with an insertion of a 748‐bp sequence (Figure 1a). We deposited this 748‐bp sequence specific to *nic2‐1* in GenBank at NCBI (accession no. LC677316). We retrieved only a few short sequences from *Nicotiana* species when using this 748‐bp sequence as a query for the BLAST search. No functional annotation could be given to the inserted sequence.

**Figure 2:**
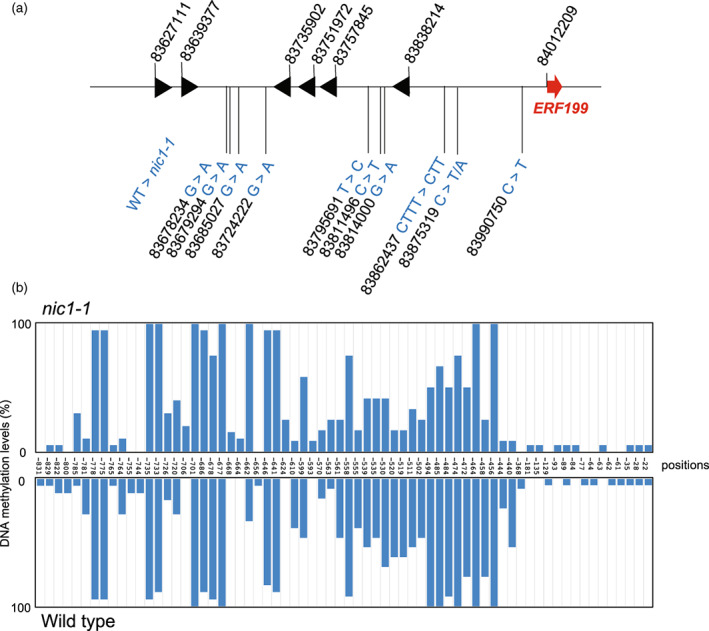
Genetic and epigenetic alterations detected in the *nic1‐1* allele. (a) Nucleotide polymorphisms at the *NIC1* locus in the *nic1‐1* allele detected by resequencing. All detected single nucleotide polymorphisms are depicted schematically, while no insertion/deletions were found in this region. Red arrow, *ERF199*; black arrowheads, other *ERF*s. The nucleotide positions of the first ATG of *ERF*s and the polymorphisms are indicated by numbers. Arrowheads indicate alterations from references. (b) DNA methylation in the 5′ flanking region of *ERF199*. DNA methylation levels (%) were examined in the wild type (WT) and the *nic1‐1* mutant in the Burley21 background using bisulfite sequencing. All positions (counted from the first ATG) of methylated cytosines are indicated.

The *NIC1* locus is delimited to a region corresponding to an *ERF199*‐containing gene cluster on chromosome 7 (Qin et al., 2021; Sui et al., 2020). All single nucleotide polymorphisms detected in *nic1‐1* around the locus during resequencing are shown in Figure 2(a), while no insertions/deletions are there. We identified no polymorphisms in regions near the *ERF* genes (between 0 bp and 10 kb). We identified two genomic regions far downstream of *ERF199* that are deleted in *nic1‐1* (Figure 1b). We determined their exact positions and sizes by sequencing the genomic fragments obtained by PCR amplification using indicated primer pairs (DF4 and DR4, LDF3 and LDR4) across the predicted deleted regions (Figure 1b). We determined that the two deleted genomic regions entail a 2907‐bp deletion between 84 057 832 and 84 060 740 bp and a 412‐kb deletion between 85 948 920 and 86 360 707 bp on chromosome 7.

To explore potential epigenetic alterations in *nic1‐1*, we determined DNA methylation levels in the roots over the 5′ flanking region of *ERF199* using bisulfite sequencing. We detected much lower methylation levels in the proximal promoter (up to 400 bp upstream of the first ATG) than in the distal region in both the wild type and the *nic1‐1* mutant (Figure 2b). We observed no evident increase in the degree of epigenetic modifications in the *nic1‐1* mutant compared with the wild‐type control (Figure 2b).

### Identification of novel low‐nicotine tobacco lines and their genotypes

Leaf alkaloid profiles were reported for 1091 tobacco introduction (TI) lines maintained by the US *Nicotiana* Germplasm Collection (Sisson & Saunders, 1982). To expand the genetic resources of low‐nicotine phenotypes, we obtained seeds for 37 lines and confirmed that 11 of them were low‐nicotine lines based on the nicotine levels (<0.2 mg/g dry weight [DW] in Experiment 1, <1 mg/g DW in Experiment 2) in the leaves of in‐house‐grown plants (Figure S2). All the lines selected here were also evaluated as being low‐alkaloid accessions in an independent study (Burner et al., 2022).

**Figure 3:**
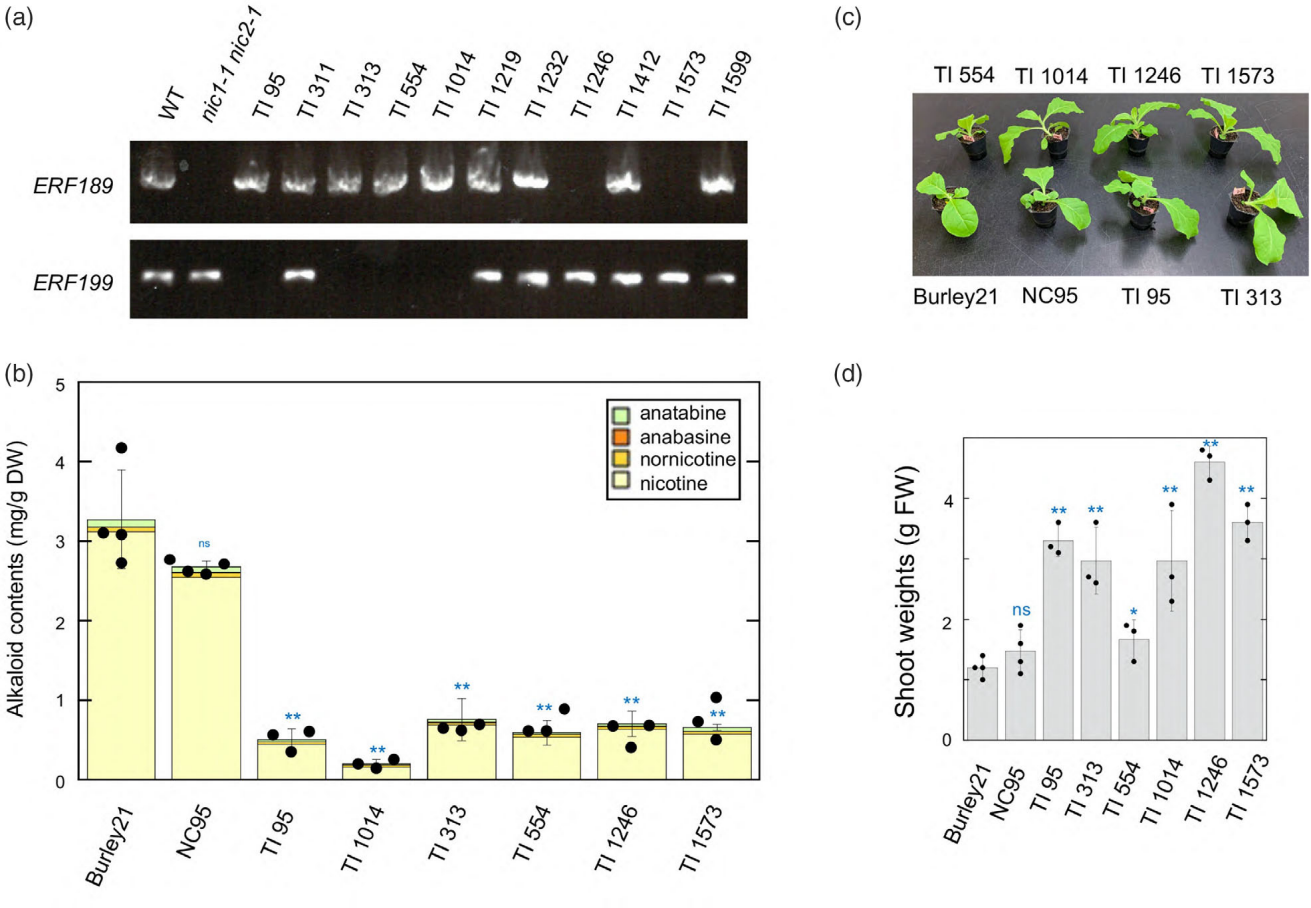
Low‐nicotine tobacco lines from the US *Nicotiana* Germplasm Collection. (a) *ERF189* and *ERF199* presence/absence was examined by genomic polymerase chain reaction analysis in tobacco introduction (TI) lines with low‐nicotine contents (Figure S2). The genotypes in the wild type (WT; Burley21) and *nic1‐1 nic2‐1* (LA Burley21) were examined as well. (b) Alkaloid contents in the leaves from 4‐week‐old plants of the six lines, in which either *ERF189* or *ERF199* is absent (Figure 1a). WT Burley21 and NC95 were included. Black dots represent total alkaloid contents of individual biological replicates, while error bars indicate their SD. DW, dry weight. (c) Images of plants used for the measurement of alkaloid contents shown in (b). (d) Fresh weights (FW) of shoots. Error bars represent the SD of the biological replicates. Significant differences relative to the Burley21 control were determined using Student's *t*‐tests in (b, d); *P < 0.05, **P < 0.01. ns, not significant.

To identify the genes controlling nicotine contents in these 11 lines, we amplified the *ERF189* and *ERF199* genes by PCR. We discovered that either *ERF* is absent in six lines, which we studied next; notably, no single line lacked both *ERF*s (Figure 3a). We determined the alkaloid contents of these six lines in the leaves of 4‐week‐old plants (Figure 3b,c). However, the measured alkaloid contents of these lines were too variable to allow reliable comparisons (Figure 3b), possibly reflecting the divergence of genetic backgrounds (Figure 3d).

Lines TI1246 and TI1573 contained *ERF199* but lacked *ERF189* (Figure 3a), similar to the *nic1‐1 nic2‐1* double mutant. We first examined whether these lines harbor the *nic1‐1* and *nic2‐1* alleles using PCR analyses. Indeed, TI1246 and TI1573 contained both the *nic1‐1* and *nic2‐1* alleles, as evidenced by the amplification of genomic fragments of identical sizes with the primers flanking the deleted regions (Figure S3a,b). Moreover, and consistent with known patterns of gene expression in the *nic1‐1 nic2‐1* genotype (Hibi et al., 1994; Qin et al., 2021; Shoji et al., 2010; Sui et al., 2020), relative *ERF199* transcript levels in TI1246 and TI1573 were 53% and 37% those of the wild type, respectively (Figure S3c). We also observed the expression of the metabolic genes *PMT* and *A622*, which were also downregulated 13–23% of their levels in the wild type (Figure S3c).

**Figure 4:**
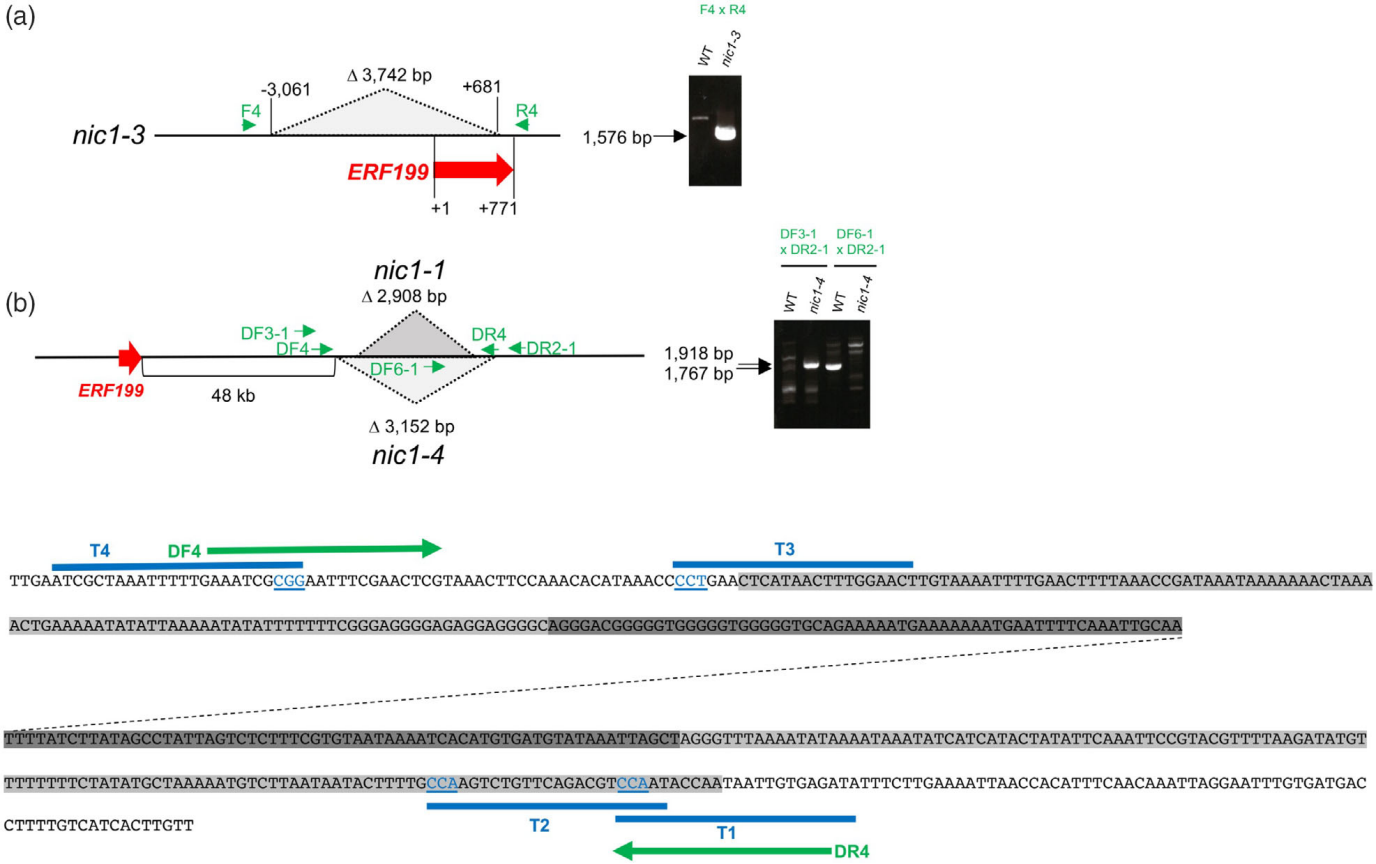
New mutant alleles *nic1‐3* and *nic1‐4*. (a) The *nic1‐3* allele harbors a 3742‐bp deletion. The genomic structure is depicted schematically. Green arrows indicate the primers used for polymerase chain reaction (PCR) analysis, and the red arrow indicates the *ERF199* coding region. The deletion in *nic1‐3* was detected by PCR amplification of a fragment of 1576 bp with F4 and R4 primers. WT, wild type. (b) The mutant allele *nic1‐4* was generated through CRISPR/Cas9‐mediated removal of a genomic sequence, including a region deleted in *nic1‐1*. Schematic diagrams of the genomic structures of *nic1‐1* and *nic1‐4* alleles. Green arrows indicate primers used for PCR, and the red arrow indicates the *ERF199* coding region. The deletion was detected by PCR amplification of two fragments (1918 bp in *nic1‐4* and 1767 bp in WT) with the indicated primers. At the bottom, sequences deleted in *nic1‐1* and *nic1‐4* are shown in dark and light gray, respectively, while the positions of the primers and target sequences (T1–T4) used in editing are shown with green arrows and blue lines, respectively. The PAM trinucleotides (blue) in the target sequences are underlined. The dashed line indicates an omitted region of the sequence.

The tobacco varieties TI95, TI313, TI554, and TI104 lacked *ERF199* (Figure 3a). As described above, we delimited the genomic regions deleted in these lines by PCR analyses using primers designed around *ERF199*. We determined that TI95, TI313, TI554, and TI1041 harbored deletions of <1300, <16, <1300, and <1640 kb, respectively (Figure S4). In particular, the TI313 lines carried a 3742‐bp deletion, which included the promoter and coding regions, excluding the last 90‐nt portion (Figure 4a). We designated the allele defined by this deletion in TI313 as *nic1‐3* (Figure 4a). To prepare a homozygous *nic1‐3* mutant with a clear background, we backcrossed T313 three times to tobacco Petit Havana SR‐I and confirmed that the resulting lines after the second backcross do not harbor a 5‐bp deletion in *MYC2a* recently reported to be present in TI313 (Burner et al., 2022).

### Characterization of the allele *nic1‐4* generated by CRISPR/Cas9‐mediated removal of a genomic region

To assess the contribution of the 2907‐bp deletion in *nic1‐1* that was 48 kb downstream of *ERF199* (Figure 1b), to the low‐nicotine phenotype, we generated a new allele by removing the corresponding genomic region via CRISPR/Cas9‐mediated genome editing. To this end, we selected four target sequences, T1–T4 (Figure 4b), located on both sides of the deletion, to construct a binary vector intended to induce double‐strand breaks at the target sites. These breaks were expected to undergo non‐homologous end‐joining repair resulting in the deletion of the genomic region between the targets. We introduced the binary vector into tobacco Petit Havana SR‐1 by Agrobacterium (*Agrobacterium tumefaciens*)‐mediated leaf disc transformation. We selected primary transgenic plants (T_0_ generation) based on drug resistance carried by the vector and tested genomic DNA by PCR analyses using primers designed to target both sides of the expected breakpoints (Figure 4b). Among the >20 plants examined, only two gave clear amplicons that were identical and thus were considered likely to originate from the same transformation event. Sequencing of the amplified fragment revealed a deletion of 3152 bp that overlaps with the interval defined by the 2907‐bp deletion in *nic1‐1*, as intended (Figure 4b). The newly generated allele partially mimicked the naturally occurring *nic1‐1* (see below) and was named *nic1‐4* (Figure 4b).

### Alkaloidal phenotypes in different *NIC1* and *NIC2* genotypes

**Figure 5:**
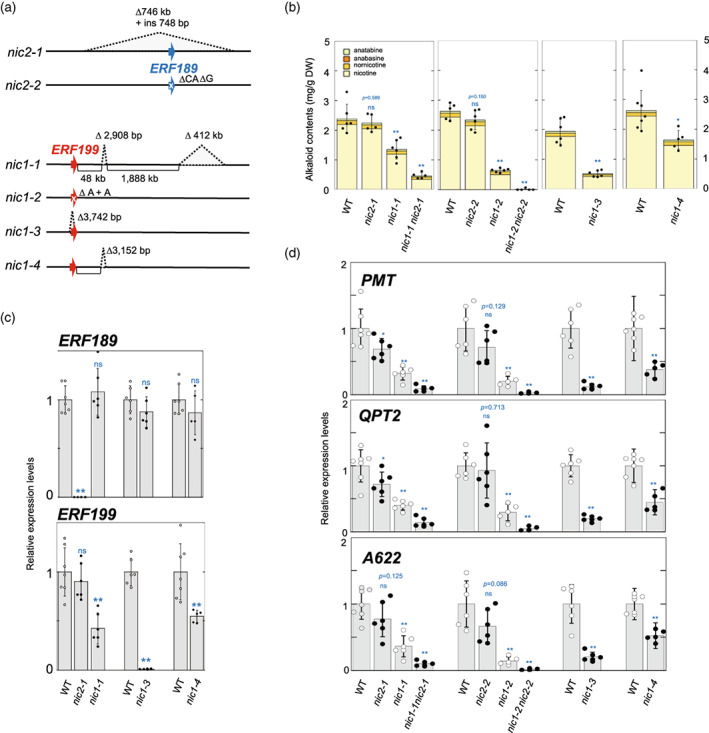
Alkaloid contents and gene expression levels in different *NIC1* and *NIC2* genotypes. Most expanded leaves and roots from 4‐week‐old plants were used for alkaloid and reverse transcription–quantitative polymerase chain reaction (RT‐qPCR) analyses. (a) Schematic diagrams of the genomic structures of the *nic1* and *nic2* mutant alleles. Blue arrow, *ERF189* coding region; red arrow, *ERF199* coding region. (b) Alkaloid contents in different genotypes. Black dots represent total alkaloid contents of individual biological replicates, while error bars indicate their SD. WT, wild type. (c) Relative expression levels of *ERF189* and *ERF199* in *nic2‐1, nic1‐1, nic1‐3*, and *nic1‐4*, and (d) those of *PMT*, *QPT2*, and *A622* in various *NIC* genotypes, as determined by RT‐qPCR. Error bars indicate the SD of the biological replicates. Expression levels in WT controls were set to 1. Significant differences relative to the WT controls were determined by Student's *t*‐tests in (b–d); *P < 0.05, **P < 0.01. *P*‐values are indicated in (b, d), when the differences were not significant (ns).

Six mutant alleles at the *NIC1* and *NIC2* loci, including both naturally occurring and generated via genome editing, have been identified through this and other studies (Figure 5a) (Hayashi et al., 2020; Hibi et al., 1994). We thus evaluated (or re‐evaluated) the relative effects of each allele on alkaloid contents and associated gene expression under the same experimental conditions. Accordingly, we collected the most expanded leaves and roots from 4‐week‐old plants for alkaloid and reverse transcription (RT)‐quantitative (q)PCR analyses, unless mentioned otherwise, using homozygous plants for each allele as described in Experimental procedures. We analyzed the apparent loss‐of‐function alleles *nic1‐2* and *nic2‐2*, which were present in an *erf189 erf199* double knockout line K2 generated in a previous study (Hayashi et al., 2020). The induced deletions (1‐bp deletion [A] in *nic1‐2*, and 2‐bp deletion [CA] and 1‐bp deletion [G] in *nic2‐2*) and insertion (1‐bp insertion [+A] in *nic1‐2*) are expected to affect ERF functions negatively by causing frameshifts, which are presumed to result in protein truncation with a premature stop codon in *nic2‐2* and substitutions of critical amino acid residues in *nic1‐2* (Hayashi et al., 2020) (Figure S5). We separated the *nic1‐2* and *nic2‐2* alleles by generating an F_2_ segregating population obtained by backcrossing the K2 line to tobacco Petit Havana SR‐I.

The *nic2‐1* and *nic2‐2* mutant alleles are thought to cause complete loss of ERF189 function and are thus likely null alleles (Figure 5a). As previously observed for *nic2‐1* (Hibi et al., 1994; Legg & Collins, 1971), the effect of both *nic2‐1* and *nic2‐2* on alkaloidal phenotypes was quite limited. Although the mean values for alkaloid contents and gene expression levels were slightly lower in all examined cases in the mutant background relative to their respective wild‐type controls, we only observed significant reductions (*P* < 0.05) in the expression levels of *PMT* and *QPT2* in *nic2‐1* (Figure 5b,d).

The four *NIC1* mutant alleles *nic1‐1*, *nic1‐2*, *nic1‐3*, and *nic1‐4* were characterized by variable effects on the alkaloidal phenotype (Figure 5b,d, Figure S6). Both *nic1‐2* and *nic1‐3* alleles are considered to be null and caused significant and severe drops in both alkaloid accumulation (Figure 5b; *P* < 0.05) and gene expression levels (Figure 5d), only reaching 25–27% and 12–29% of the levels in their wild‐type controls, respectively.

By contrast, the *nic1‐1* and *nic1‐4* alleles, which both carried a genomic deletion of equivalent size at similar positions downstream of *ERF199* (Figure 4b, Figure 5a), had more moderate but significant effects on alkaloid accumulation (57–73% of wild‐type levels) (Figure 5b) and gene expression levels (32–52% of wild‐type levels) (Figure 5d). Moreover, these observed effects were intermediate between the mild alterations seen in *nic2* alleles and the severe phenotypes of *nic1* null alleles. The nearly comparable strength of *nic1‐1* and *nic1‐4* was also reflected in their similar attenuation of *ERF199* expression (Figure 5c). We observed a comparable moderate influence of *nic1‐4* on the alkaloid contents in the leaves and roots and associated gene expression in the roots from plants at different growth stages relative to the wild type (Figure S6).

In line with previous reports (Hayashi et al., 2020; Hibi et al., 1994), alkaloid levels (Figure 5b) and transcript levels of metabolism‐related genes (Figure 5d) reached 20% and 9–14% of wild‐type levels in *nic1‐1 nic2‐1* and 1% and 2–5% of wild‐type levels in *nic1‐2 nic2‐2*, respectively. Although the effects of *nic2‐1* and *nic2‐2* single mutations on the phenotypes were relatively slight, both double mutants exhibited a more pronounced phenotype than their respective *nic1* single mutants, indicating that the loss of ERF189 function becomes clearer when ERF199 function is compromised or completely absent. We hypothesize that the clear differences between the two double mutants reflect the greater severity of *nic1‐2* compared with *nic1‐1*, given the nearly identical phenotypic effects of *nic2‐1* and *nic2‐2*.

To clarify whether the loss or reduced activity of one of the ERFs has any influence on the expression of other *ERF* genes, we examined the expression levels of *ERF189* and *ERF199* by RT‐qPCR in the *nic2‐1, nic1‐1, nic1‐3*, and *nic1‐4* mutants (Figure 5c). We detected no or very low abundance for *ERF189* transcripts in *nic2‐1* or for *ERF199* transcripts in *nic1‐3*, in agreement with the genomic deletions harbored by each mutant and the use of highly specific primers (Figure 5c). We observed no significant alterations for *ERF189* or *ERF199* expression in *nic1* or *nic2* mutants relative to the wild‐type controls, respectively (Figure 5c), indicating an absence of reciprocal regulation between ERF189 and ERF199.

### Functional equivalence of ERF189 and ERF199

**Figure 6:**
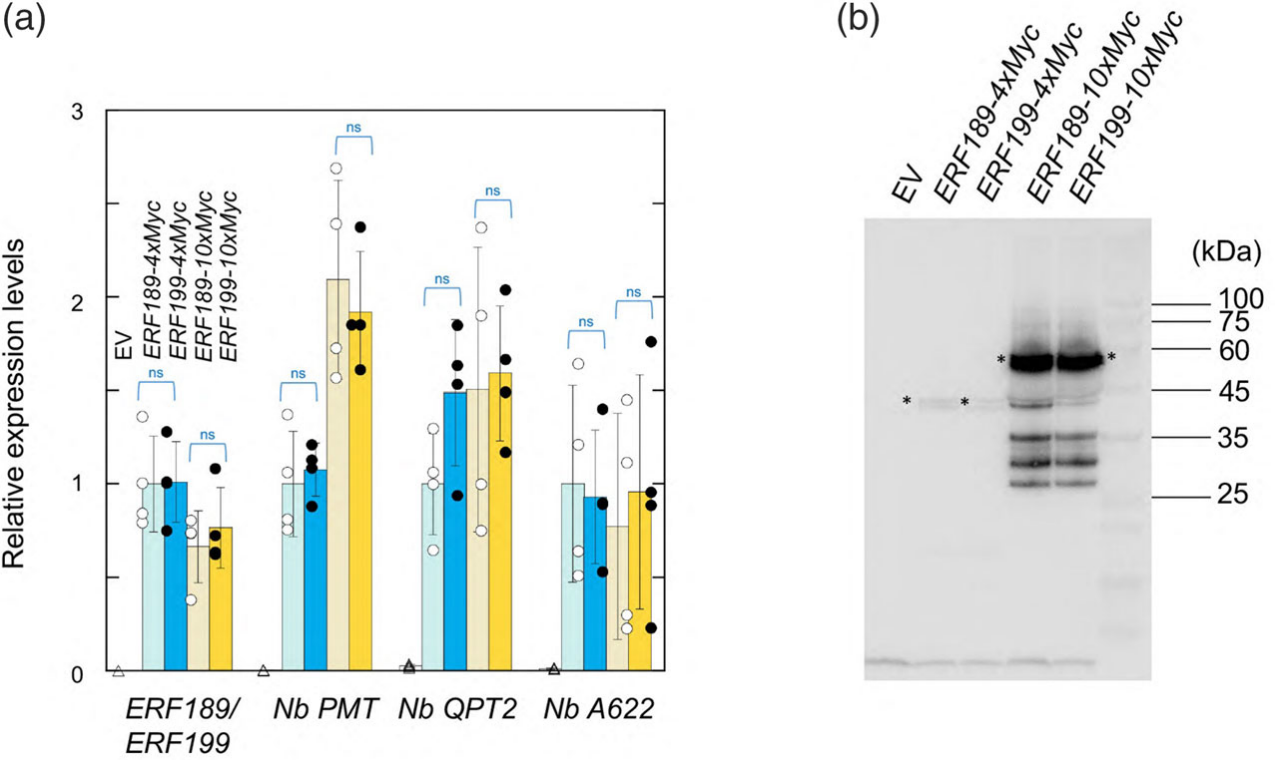
Transient overexpression of *ERF189* and *ERF199* genes in *Nicotiana benthamiana* leaves. The tobacco *ERF*s with *4 × Myc* or *10 × Myc* tag sequences at their 3′ ends were overexpressed under the control of the CaMV 35S promoter. (a) Relative expression levels of nicotine biosynthesis genes, as determined by RT‐qPCR. *ERF189* and *ERF199* were amplified together with primers designed to target a conserved region in both genes. Expression levels in leaves were set to 1, where *ERF189‐4 × Myc* was overexpressed. Error bars indicate the SD of the biological replicates. Significant differences were determined between the indicated samples by Student's *t*‐tests. ns, not significant. (b) Accumulation of Myc‐tagged ERF189 and ERF199 fusion proteins, as determined by immunoblot analysis. Major detected bands are indicated with asterisks.

As the phenotypic effects of the *nic1* null allele were much stronger than those of the *nic2* null allele were (Figure 5b,d), we explored the possible functional differences between ERF189 and ERF199 as transcriptional regulators. Ectopic overexpression of *ERF189* induces nicotine biosynthesis in the leaves of *Nicotiana* plants (Hayashi et al., 2020). We transiently overexpressed constructs encoding each protein fused to a Myc‐tag (as 4 [4 × Myc] or 10 [10 × Myc] copies) via Agrobacterium‐mediated infiltration in *Nicotiana benthamiana* leaves and analyzed the expected transactivation of the downstream metabolic genes involved in nicotine biosynthesis (Figure 6). The expression levels of the *ERF189* and *ERF199* effectors and the accumulation of their proteins were nearly identical (Figure 6). However, their molecular weights detected by immunoblot analysis did not match their expected sizes of 32 kDa (4 × Myc) and 40 kDa (10 × Myc), suggesting the presence of unknown post‐translational modifications (Figure 6b). Importantly, the transcript levels of the *N. benthamiana* metabolic genes *PMT*, *QPT2*, and *A622* were all upregulated and reached comparable levels in *ERF189*‐ and *ERF199*‐overexpressing leaves (Figure 6a), suggesting that the two homologous ERFs have nearly equivalent functions.

In tobacco roots, *ERF189* and *ERF199* transcripts accumulate to similar levels (Kajikawa et al., 2017; Shoji et al., 2010). In line with this observation, a meta‐analysis of multiple transcriptome datasets revealed only slightly higher expression for *ERF199* compared with *ERF189* in tobacco roots (Figure S7).

## DISCUSSION

### Mild effects of the *nic2* null mutation on alkaloid phenotypes

The tobacco varieties TI1246 and TI1573 carry the same *nic2‐1* allele (Figure 1a) previously identified in LA Burley 21 (Legg et al., 1970), which was derived from the original strain of Cuban cigar tobacco (Valleau, 1949). While data are not available for TI1573, TI1246 is of the oriental type and native to Europe (Sisson & Saunders, 1982), which is clearly phylogenetically distinct from cigar tobacco. In all cases, *nic2‐1* was present in combination with *nic1‐1* (Valleau, 1949) (Figure 1a,b), indicating that the low‐nicotine phenotype emerges only when the weak *nic2‐1* and the moderate *nic1‐1* alleles combine. This phenotypic magnification in the double mutants was also evident in the case of the *nic1‐2 nic2‐2* double mutant compared with the *nic1‐2* single mutant (Figure 5b,d). These results indicate that the loss of ERF189 function becomes apparent only in genetic backgrounds where the activity of the functionally redundant ERF199 is lost or diminished.

The loss of ERF189 was also demonstrated in other tobacco accessions in an independent study (Burner et al., 2022). It would be worth examining whether these other accessions harbor *nic2‐1* or other *nic2* alleles and what mutations must accompany *nic2* to evoke low‐nicotine phenotypes.

Although *nic2‐1* and the CRISPR/Cas9‐generated allele *nic2‐2* were structurally different, both completely lost functional ERF189 (Figure 5a) and have comparative negative but limited impacts on nicotine phenotype (Figure 5b,d). This result indicates that the alkaloidal phenotype is not specific to either allele and that the loss of a genomic region apart from *ERF189*, including other *ERF*s found lost in *nic2‐1*, does not significantly contribute to the nicotine phenotype. These findings are in line with the notion that no other ERFs outside of ERF189 and ERF199 play a major role in the regulation of nicotine biosynthesis (Hayashi et al., 2020).

It has not been determined why the loss of ERF189 function has a much milder effect on the alkaloid phenotype than that of ERF199. The homologous genes *ERF189* and *ERF199* in the natural allotetraploid tobacco species are considered derived from the parental *Nicotiana* diploids *N. tomentosiformis* and woodland tobacco (*N. sylvestris*), respectively (Shoji et al., 2010). As alkaloid accumulation is comparable between the two wild species (Saito et al., 1985), *N. tomentosiformis* and *N. sylvestris* may have similar abilities to produce alkaloids. Therefore, it is reasonable to hypothesize that ERF189 and ERF199 are transcriptional regulators of similar activity. In line with this notion, transient transactivation analysis in *N. benthamiana* indicated nearly equal functionalities of these ERFs (Figure 6); note that the encoding constructs were ectopically overexpressed from a constitutive promoter, and therefore it is not certain how exactly the results recapitulate native functionalities of the factors. In this overexpression system, both *ERF*s had a similar accumulation of transcripts and proteins (Figure 6), indicating no significant difference in their stability. Consistent with previous reports (Kajikawa et al., 2017; Shoji et al., 2010), a meta‐analysis of transcriptome data indicated that *ERF189* and *ERF199* expression levels are not very different at the transcript level in tobacco roots, where they direct alkaloid biosynthesis (Figure S7). Further studies should define the molecular sizes of the fusion proteins observed in immunoblot analysis (Figure 6b) and possible long‐range gene regulation, as discussed below, to define the mechanisms of ERF function and regulation and provide insights into the distinct phenotypic consequences of either loss of function.

### Phenotypic consequences of different *nic1* mutant alleles

A deletion of *ERF199* has been detected in nine tobacco germplasms with low‐nicotine traits: TI95, TI313, TI554, and TI1041 analyzed in this study and TI508, TI516, TI527, TI1211, and TI785 reported additionally by Burner et al. (2022). The positions and sizes of these individual *ERF199* deletions were mostly distinct between the examined lines (Figure 4a, Figure S4), suggesting the occurrence of multiple alleles that possibly arose independently, indicating that *ERF199* may be a mutational hotspot for low‐nicotine traits. In contrast to *ERF199*, the loss of ERF189 confers a modest effect on the same phenotypes and only evoked a strong phenotype in double mutant states. We characterized the representative allele, *nic1‐3*, found in TI313, in detail after removing background mutations via backcrossing. The extent of decrease in alkaloid contents caused by *nic1‐3* and *nic1‐2*, which also leads to the complete loss of ERF199 function, was nearly equivalent to that seen in the *nic1‐1 nic2‐1* double mutant (Figure 5b,d). These results are consistent with the fact that the lines lacking *ERF199* were selected along with lines of the *nic1‐1 nic2‐1* genotype with a similar alkaloid accumulation range (Figure 3b, Figure S2) (Burner et al., 2022).

We obtained moderate but significant effects on the alkaloid phenotype produced by the *nic1‐1* and *nic1‐4* alleles (Figure 5, Figure S6), which carried similar genomic deletions distal to *ERF199* (Figure 4b). The expression of *ERF199* was similarly downregulated in *nic1‐1* and *nic1‐4* (Figure 5c, Figure S6) (Qin et al., 2021; Shoji et al., 2010; Sui et al., 2020). This shared deletion between the two alleles is likely to be responsible for the observed phenotype. We did not notice clear functionally relevant elements or motifs within the deleted sequence, possibly stemming from a rudimentary understanding of the elements involved in long‐range gene regulation. Moreover, we identified no stretch of homologous sequences in the genomic region around *ERF189* at the *NIC2* locus, suggesting this mode of gene regulation is specific to the *NIC1* locus. How the deleted genomic sequence, apart from *ERF199* itself, regulates nicotine‐controlling genes is not known, but it may entail chromatin‐based mechanisms (Grosveld et al., 2021; Jain & Garg, 2021; Miele & Dekker, 2008). Notably, we detected no significant increase in DNA methylation in *nic1‐1* within the *ERF199* promoter region (Figure 2b), indicating that the epigenetic regulation is not involved in this process.

Given that the *nic1‐1* allele occurs naturally with deletions only at positions distal to *ERF199*, other *nic1* alleles may also contribute to low‐nicotine traits but may not be detected by simple PCR amplification of *ERF199*. The screening of varieties based on *ERF199* expression levels may expand the allelic repertoire available at the *NIC1* locus, which would provide clues to elucidating the unknown mechanisms of *ERF199* regulation.

### Breeding low‐nicotine tobacco

Nicotine is a highly addictive stimulant that contributes to strong dependence on tobacco products. Reducing nicotine levels in tobacco is a key measure to promote smoking cessation and limit possible environmental contamination due to this highly toxic chemical, which is an important issue in the mass cultivation of tobacco. The recommended nicotine contents in filter cigarettes are <0.4 mg g^−1^, according to the World Health Organization (WHO, 2015); however, no commercial tobacco cultivar reaches such a low level of nicotine consistently. Several attempts to reduce tobacco alkaloid contents through transgenic and editing approaches have been reported (Hayashi et al., 2020; Hidalgo Martinez et al., 2020; Lewis et al., 2015). Nevertheless, because of regulatory and intellectual property concerns associated with genetic engineering and editing, expanding the repertoire of naturally occurring genetic variants that might be applicable to low‐nicotine tobacco breeding has become increasingly valuable (Lewis et al., 2020). The elucidation at the sequence level of an allelic series of *nic1* and *nic2* mutations, which have major effects on alkaloid accumulation, will pave the way for PCR‐based genotyping at these loci for tobacco breeding, as demonstrated in this study.

We did not identify tobacco varieties lacking both *ERF189* and *ERF199* in the 11 lines tested in this study (Figure 3a) (Burner et al., 2022). It is unclear whether the absence of such natural mutants has any meaning. Nevertheless, the presence of single loss‐of‐function mutants for either *ERF* allows the generation of such allelic combinations through simple genetic crosses and may produce ultra‐low nicotine levels. One possible explanation for the absence of these double mutants in the collection is the metabolic imbalance induced by the nearly complete shutdown of the nicotine pathway with the loss of both ERFs. This imbalance was observed in *erf189 erf199* double knockout lines generated by genome editing (Hayashi et al., 2020), which may suffer growth penalties or other limitations for survival in natural environments.

In summary, we revealed an allelic series at the *NIC1* and *NIC2* loci across a number of tobacco germplasms and highlighted the significance of *ERF189* and *ERF199* in the regulation of the nicotine pathway (Hayashi et al., 2020). Although not the focus of this study, it is clear that other as yet undefined factors also contribute to low‐nicotine phenotypes (Burner et al., 2022), as alkaloid accumulation is under complex genetic control. It would be valuable to study further the low‐nicotine tobacco resources with the knowledge gained from the defined *nic1* and *nic2* mutations from this study.

## EXPERIMENTAL PROCEDURES

### Plant materials

Surface‐sterilized seeds of tobacco (*N. tabacum*) were germinated and grown to the seedling stage on half‐strength Gamborg's B5 medium solidified with 0.7% (w/v) agar and supplemented with 3% (w/v) sucrose. Two‐week‐old seedlings were transferred to soil in pots and grown to maturity. Wild‐type, *nic2‐1*, *nic1‐1*, and *nic1‐1 nic2‐1* genotypes in the Burley21 background (Legg et al., 1970; Legg & Collins, 1971) were obtained from the USDA, whereas all other tobacco lines, including those of the US *Nicotiana* Germplasm Collection (Sisson & Saunders, 1982), were obtained from Japan Tobacco.

Except for a mutant series for *nic1‐1* and *nic2‐1* in the Burley21 background, all plants harboring certain mutant alleles were crossed with wild‐type tobacco Petit Havana SR‐I. In subsequent segregating generations, homozygous plants for each allele were selected using PCR‐based genotyping. The selected plants were self‐pollinated, and their progeny were analyzed. Plants derived from segregants carrying the wild‐type alleles were used as wild‐type controls.

The low‐alkaloid TI313 line carrying *nic1‐3* was backcrossed three times to Petit Havana SR‐I to remove most of the TI313 background. We confirmed that a 5‐bp deletion at *MYC2a* in TI313, which was reported during the course of this study (Burner et al., 2022), was not present through the repeated crossings by checking the sequences of the plants obtained after the second backcross.

### Plant transformation

A binary vector for CRISPR/Cas9‐mediated editing was constructed based on the pMg237‐2A‐GFP plasmid by PCR amplification followed by Golden Gate cloning using the restriction enzyme *Bsa*I as described (Nakayasu et al., 2018). Four target sequences (T1, TATCTCACAATTATTGGTATTGG; T2, ATTGGACGTCTGAACAGACTTGG; T3, AGTTCCAAAGTTATGAGTTCAGG; T4, CCGCGATTTCAAAAATTTAGCGAT) were inserted into single guide RNA (sgRNA) cassettes. The construct was introduced into Agrobacterium (*A. tumefaciens*) strain EHA105 by the heat shock method. Leaf disc transformation was performed to generate transgenic tobacco lines (Horsch et al., 1985). Transgenic plants from the T_0_ generation were selected based on drug resistance.

### Genome resequencing

Genomic DNA was extracted with the cetyltrimethylammonium bromide method (Murry & Thompson, 1980). DNA sequencing libraries were prepared starting from 100 ng of isolated DNA using the Ovation Ultralow V2 DNA‐Seq Library Preparation kit (Tecan, Männedorf, Switzerland), normalized to 10 nm, multiplexed, and clustered on HiSeq 3000/4000 PE flow cells using HiSeq 3000/4000 PE Cluster kits (Illumina, San Diego, CA, USA). Sequencing was performed on an Illumina HiSeq 4000 system using an Illumina HiSeq 3000/4000 SBS kit (300 cycles).

Raw reads were mapped to a chromosome‐anchored tobacco K326 reference genome (registration in progress) using Minimap2 (v2.16‐r922), and duplicates were removed using samblaster (v0.1.24). samtools (v1.9) was used to sort the read alignments and store them in BAM format, while bedtools (v2.28.0) was used to determine genome coverage.

Polymorphisms around the *NIC1* locus were detected by analyzing mapped sequencing reads from Burley 21 (wild type), LI Burley 21 (*nic1‐1*), and LA Burley 21 (*nic1‐1 nic2‐1*) using bcftools (v1.9), filtering out variants with a call quality <30 or a read depth <20. The obtained variants were further filtered to retain only those that were also detected when comparing NC95 (the wild type) and LAFC53 (*nic1‐1 nic2‐1*).

### Genomic PCR analysis

Genomic DNA was used as the template for PCR with Ex Taq DNA polymerase (Takara, Kyoto, Japan). The primer sequences are listed in Table S1. The PCR thermal program details of each primer pair are available upon request. The amplified fragments were sequenced after cloning into the T‐vector pMD20 (Takara). For genotyping of *nic1‐2* and *nic2‐2* derived from the *erf189 erf19*9 double knockout line K2 (Hayashi et al., 2020), PCR products were separated on 10% polyacrylamide gels after digestion with restriction enzymes *Ava*II and *Bsr*I (Figure S5); the other amplicons were separated on 0.8% (w/v) agarose gels. The GelRed Prestain Plus loading dye (Biotium, Fremont, CA, USA) was used to visualize the DNA fragments.

### RT‐qPCR analysis

Total RNA was isolated from samples ground in liquid nitrogen using an RNeasy kit (Qiagen, Venlo, Netherlands). Total RNA was then converted to first‐strand cDNA using the SuperScript IV reverse transcriptase (Thermo Fisher Scientific, Waltham, MA, USA) and an oligo(dT) primer. The cDNA templates were amplified using a StepOnePlus Real‐Time PCR system (Thermo Fisher Scientific) and the Fast SYBR Green Master Mix (Thermo Fisher Scientific). The primer sequences are listed in Table S2. The thermal program was 20 sec at 95°C followed by 40 cycles of 3 sec at 95°C and 30 sec at 60°C. *EF1α* was used as the reference gene. Each assay was performed at least three times.

### Bisulfite sequencing

Genomic DNA (500 ng) was modified using the EpiSight Bisulfite Conversion kit v2 (Fujifilm, Tokyo, Japan). Bisulfite‐treated DNA was amplified by PCR using the primers listed in Table S3. The PCR products were cloned into pMD20 (Takara), and over 12 clones for each amplicon/genotype combination were sequenced to estimate the methylation levels (%).

### Alkaloid contents analysis

A dry leaf or root sample of 10 mg was homogenized and soaked in 1 ml of 0.1 N H_2_SO_4_. After sonication and centrifugation, 1 ml of the supernatant was mixed with 0.1 ml of 25% (w/v) NH_4_OH, loaded on to an Extrelut‐1 column (Merck, Darmstadt, Germany), and eluted with 6 ml of chloroform. The extract was dried at 30°C using an E1‐25 evaporation head (Taitec, Koshigaya, Saitama, Japan) and dissolved in ethanol containing 0.1% (v/v) dodecane. Tobacco alkaloids were separated and quantified with a gas–liquid chromatograph (GCMS‐TQ8040; Shimadzu, Kyoto, Japan) equipped with an InertCap 5MS/NP capillary column (GL Science, Tokyo, Japan) using a thermal gradient of 80°C for 2 min, 30°C/min to 320°C, and 320°C for 2 min.

### Agrobacterium‐mediated transient overexpression

The full‐length coding regions of *ERF189* and *ERF199* were amplified by PCR and cloned into pENTR/D‐TOPO (Thermo Fisher Scientific). The cloned sequences were transferred to Gateway binary vectors pGWB417 and pGWB420 (Nakagawa et al., 2007) through a reaction catalyzed by LR Clonase II (Thermo Fisher Scientific). The resulting vectors for overexpression with an added Myc‐tag and the *p19* vector for suppression of silencing were used as follows. Suspensions (OD_600_ = 0.25) of Agrobacterium strain GV3101 harboring each overexpression vector and *p19* vector were combined at a ratio of 7:3 and infiltrated into the topmost fully expanded leaves of 6‐week‐old *N. benthamiana* plants (Shoji, 2018). Transcript and protein levels were analyzed 2 days after infiltration.

### Immunoblot analysis

Leaves were frozen in liquid nitrogen and ground into a fine powder. The samples (100 mg fresh weight) were sonicated in 250 μl phosphate buffered saline buffer (10 mm sodium phosphate, pH 7.5, 0.8% [w/v] NaCl, and 0.02% [w/v] KCl) containing 1 mm phenylmethyl sulfonyl fluoride and 5 mm dithiothreitol using a Sonifier 250 (Branson, St. Louis, MO, USA). After centrifugation of the homogenates at 20 000 ***g*** at 4°C for 5 min, the soluble protein extracts in the supernatant were mixed with an equal volume of EzApply (Atto, Tokyo, Japan) and boiled at 95°C for 10 min. The samples (10 μl per lane) were separated by sodium dodecyl sulfate–polyacrylamide gel electrophoresis (12.5% [w/v]) gels and transferred on to a polyvinylidene fluoride membrane of the Q Blot kit M (Atto) using the NA‐1512 transfer system (Nippon Eido, Tokyo, Japan). The EzProtein Ladder (Atto) was used as the molecular size marker. The membranes were blocked and incubated with antibodies in EzBlock BSA, and a 10‐fold diluted solution of EzTBS (Atto) was used to rinse the membranes. The anti‐Myc tag polyclonal antibody (PA1‐981; Thermo Fisher Scientific) and goat horseradish peroxidase–conjugated anti‐rabbit IgG (H + L) (31 460, Thermo Fisher Scientific) were diluted at 1:2000 and 1:10 000 as primary and secondary antibodies, respectively. Chemiluminescence was developed by incubation in ECL Western Blotting Substrate (Thermo Fisher Scientific) and detected using the ImageQuant LAS 4010 (GE Healthcare, Chicago, IL, USA).

### Meta‐analysis of *ERF189* and *ERF199* expression

Publicly available transcriptome datasets (SRR1199063, SRR1199066, SRR1199068‐SRR1199074, SRR1199121‐SRR1199125, SRR1199127‐SRR1199130, SRR1199132, SRR1199135, SRR1199197‐SRR1199200, SRR1199202, SRR1199203, SRR955761‐SRR955763, and SRR955765‐SRR955767) were used to investigate the expression levels of *ERF189* and *ERF199* in tobacco roots and other organs. Sequencing reads were filtered for quality using fastp, and the raw mapped reads per gene were obtained using STAR. Count normalization was performed using DESeq2.

## ACCESSION NUMBERS

A 748‐bp sequence found to be inserted in the *nic2‐1* mutant was deposited in GenBank under the accession number LC677316. Genome resequencing data are available under accession numbers SRR18653029–SRR18653033 from Bioproject PRJNA823906.

## AUTHOR CONTRIBUTIONS

TS and NS conceived the research plans. TS, NS, NVI, TH, and KS supervised the study. NS and SO conducted the genome resequencing and meta‐analysis of transcriptomics data. KM screened low‐nicotine accessions from the germplasm collection and delimited the deleted genomic regions in the lines missing *ERF199* by genomic PCR analyses. TS performed all of the other experiments and data analyses. TS prepared the draft manuscript with the help of NS. All the authors reviewed the contents and approved the final version of manuscript.

## CONFLICT OF INTERESTS

NVI, NS, and SO are employees of Philip Morris International. The other authors declare that they have no competing interests.

## Supporting information


**Figure S1.** Biosynthetic pathway of nicotine alkaloids in tobacco.
**Figure S2.** Leaf nicotine contents in tobacco lines from the US *Nicotiana* Germplasm Collection (Sisson & Saunders, 1982).
**Figure S3.** Lines TI1246 and TI1573 have *nic1‐1* and *nic2‐1* alleles.
**Figure S4.** Deletions of genomic regions including *ERF199* in the low‐nicotine lines.
**Figure S5.** Genotyping of *nic1‐2* and *nic2‐2* alleles generated by CRISPR/Cas9‐mediated editing.
**Figure S6.** Alkaloid contents and expression levels of nicotine biosynthesis genes in 7‐week‐old tobacco plants with the *nic1‐4* genotype.
**Figure S7.** Expression levels of *ERF189* and *ERF199* in the roots and other organs from *Nicotiana tabacum* TN90.

**Table S1.** Primers used in genomic PCR analyses.
**Table S2.** Primers used in RT‐qPCR analyses.
**Table S3.** Primers used in bisulfite sequencing.

## Data Availability

The authors confirm that the data supporting the findings of this study are available within the article and in public databases under the accession codes presented in the article. A reference genome sequence for the tobacco variety K326, for which manuscript is in preparation, is available upon request.

## References

[tpj15923-bib-0001] Burner, N. , McCauley, A. , Pramod, S. , Frederick, J. , Steede, T. , Kernodle, S.P. et al. (2022) Analyses of diverse low alkaloid tobacco germplasm identify naturally occoring nucleotide variability contributing to reduced leaf nicotine accumulation. Molecular Breeding, 42, 4. 10.1007/s11032-021-01274-5 PMC10248598 37309485

[tpj15923-bib-0002] Cappellini, F. , Marinelli, A. , Toccaceli, M. , Tonelli, C. & Petroni, K. (2021) Anthocyanins: from mechanisms of regulation in plants to health benefits in foods. Frontiers in Plant Science, 12, 748049. 34777426 10.3389/fpls.2021.748049 PMC8580863

[tpj15923-bib-0003] Chaplin, J.F. (1975) Registration of LAFC53 tobacco germplasm. Crop Science, 15, 282.

[tpj15923-bib-0004] Dewey, R.E. & Xie, J. (2013) Molecular genetics of alkaloid biosynthesis in Nicotiana tabacum. Phytochemistry, 94, 10–27. 23953973 10.1016/j.phytochem.2013.06.002

[tpj15923-bib-0005] Grosveld, F. , van Staalduinen, J. & Stadhouders, R. (2021) Transcriptional regulation by (super)enhancers: from discovery to mechanisms. Annual Review of Genomics and Human Genetics, 22, 127–146. 10.1146/annurev-genom-122220-093818 33951408

[tpj15923-bib-0006] Hayashi, S. , Watanabe, M. , Kobayashi, M. , Tohge, T. , Hashimoto, T. & Shoji, T. (2020) Genetic manipulation of transcriptional regulators alters nicotine biosynthesis in tobacco. Plant & Cell Physiology, 61, 1041–1053. 32191315 10.1093/pcp/pcaa036

[tpj15923-bib-0007] Hibi, N. , Higashiguchi, S. , Hashimoto, T. & Yamada, Y. (1994) Gene expression in tobacco low‐nicotine mutants. Plant Cell, 6, 723–735. 8038607 10.1105/tpc.6.5.723 PMC160471

[tpj15923-bib-0008] Hidalgo Martinez, D. , Payyavula, R.S. , Kudithipudi, C. , Shen, Y. , Xu, D. , Warek, U. et al. (2020) Genetic attenuation of alkaloids and nicotine content in tobacco (Nicotiana tabacum). Planta, 251, 92. 32242247 10.1007/s00425-020-03387-1

[tpj15923-bib-0009] Horsch, R.B. , Fry, J.B. , Hoffmann, N.L. , Eichholtz, D. , Rogers, S.G. & Fraley, R.T. (1985) A simple and general method for transferring genes into plants. Science, 227, 1229–1231. 17757866 10.1126/science.227.4691.1229

[tpj15923-bib-0010] Jain, M. & Garg, R. (2021) Enhancers as potential targets for engineering salinity stress tolerance in crop plants. Physiologia Plantarum, 173, 1382–1391. 33837536 10.1111/ppl.13421

[tpj15923-bib-0011] Kajikawa, M. , Hirai, N. & Hashimoto, T. (2009) A PIP‐family protein is required for biosynthesis of tobacco alkaloids. Plant Molecular Biology, 69, 287–298. 19002761 10.1007/s11103-008-9424-3

[tpj15923-bib-0012] Kajikawa, M. , Sierro, N. , Kawaguchi, H. , Bakaher, N. , Ivanov, N.V. , Hashimoto, T. et al. (2017) Genomic insights into the evolution of the nicotine biosynthesis pathway in tobacco. Plant Physiology, 174, 999–1011. 28584068 10.1104/pp.17.00070 PMC5462024

[tpj15923-bib-0013] Legg, P.D. & Collins, G.B. (1971) Inheritance of percent total alkaloids in Nicotiana tabacum L. II. Genetic effects of two loci in Burley21 x LA Burley21 populations. Canadian Journal of Genetics and Cytology, 13, 287–291.

[tpj15923-bib-0014] Legg, P.D. , Collins, G.B. & Litton, C.C. (1970) Registration of LA Burley21 tobacco germplasm. Crop Science, 10, 212.

[tpj15923-bib-0015] Lewis, R.S. , Drake‐Stowe, K.E. , Heim, C. , Steede, T. , Smith, W. & Dewey, R.E. (2020) Genetic and agronomic analysis of tobacco genotypes exhibiting reduced nicotine accumulation due to induced mutations in berberine bridge like (BBL) genes. Frontiers in Plant Science, 11, 368. 32318084 10.3389/fpls.2020.00368 PMC7147384

[tpj15923-bib-0016] Lewis, R.S. , Lopez, H.O. , Bowen, S.W. , Andres, K.R. , Steede, W.T. & Dewey, R.E. (2015) Transgenic and mutation‐based suppression of a berberine bridge enzyme‐like (BBL) gene family reduces alkaloid content in field‐grown tobacco. PLoS One, 10, e0117273. 25688975 10.1371/journal.pone.0117273 PMC4331498

[tpj15923-bib-0017] Miele, A. & Dekker, J. (2008) Long‐range chromosomal interactions and gene regulation. Molecular BioSystems, 4, 1046–1057. 18931780 10.1039/b803580f PMC2653627

[tpj15923-bib-0018] Millgate, A.G. , Pogson, B.J. , Wilson, I.W. , Kutchan, T.M. , Zenk, M.H. , Gerlach, W.L. et al. (2004) Analgesia: morphine‐pathway block in top1 poppies. Nature, 431, 413–414. 15386001 10.1038/431413a

[tpj15923-bib-0019] Murry, M.G. & Thompson, W.F. (1980) Rapid isolation of high molecular weight plant DNA. Nucleic Acids Research, 8, 4321–4325. 7433111 10.1093/nar/8.19.4321 PMC324241

[tpj15923-bib-0020] Nakagawa, T. , Suzuki, T. , Murata, S. , Nakamura, S. , Hino, T. , Maeo, K. et al. (2007) Improved gateway binary vectors: high‐performance vectors for creation of fusion constructs in transgenic analysis of plants. Bioscience, Biotechnology, and Biochemistry, 71, 2095–2100. 17690442 10.1271/bbb.70216

[tpj15923-bib-0021] Nakayasu, M. , Shioya, N. , Shikata, M. , Thagun, C. , Abdelkareem, A. , Okabe, Y. et al. (2018) JRE4 is a master transcriptional regulator of defense‐related steroidal glycoalkaloids in tomato. The Plant Journal, 94, 975–990. 29569783 10.1111/tpj.13911

[tpj15923-bib-0022] Qin, B. , Eagles, J. , Mellon, F.A. , Mylona, P. , Peña‐Rodriguez, L. & Osbourn, A.E. (2010) High throughput screening of mutants of oat that are defective in triterpene synthesis. Phytochemistry, 71, 1245–1252. 20557911 10.1016/j.phytochem.2010.05.016

[tpj15923-bib-0023] Qin, Q. , Humphry, M. , Gilles, T. , Fisher, A. , Patra, B. , Singh, S.K. et al. (2021) NIC1 cloning and gene editing generates low‐nicotine tobacco plants. Plant Biotechnology Journal, 19, 2150–2152. 34468078 10.1111/pbi.13694 PMC8541770

[tpj15923-bib-0024] Saito, K. , Noma, M. & Kawashima, N. (1985) The alkaloiod contents of sixty nicotiana species. Phytochemistry, 24, 477–480.

[tpj15923-bib-0025] Shoji, T. (2018) Analysis of the intracellular localization of transiently expressed and fluorescently labeled copper‐containing amine oxidases, diamine oxidase and *N*‐methylputrescine oxidase in tobacco, using an agrobacterium infiltration protocol. Methods in Molecular Biology, 1694, 215–223. 29080170 10.1007/978-1-4939-7398-9_20

[tpj15923-bib-0026] Shoji, T. (2020) Nicotine biosynthesis, transport, and regulation in tobacco: insights into the evolution of a metabolic pathway. In: The Tobacco Plant Genome, pp. 147–156.

[tpj15923-bib-0027] Shoji, T. & Hashimoto, T. (2011) Recruitment of a duplicated primary metabolism gene into the nicotine biosynthesis regulon in tobacco. The Plant Journal, 67, 949–959. 21605206 10.1111/j.1365-313X.2011.04647.x

[tpj15923-bib-0028] Shoji, T. , Kajikawa, M. & Hashimoto, T. (2010) Clustered transcription factor genes regulate nicotine biosynthesis in tobacco. Plant Cell, 22, 3390–3409. 20959558 10.1105/tpc.110.078543 PMC2990138

[tpj15923-bib-0029] Shoji, T. , Mishima, M. & Hashimoto, T. (2013) Divergent DNA‐binding specificities of a group of ETHYLENE RESPONSE FACTOR transcription factors involved in plant defense. Plant Physiology, 162, 977–990. 23629834 10.1104/pp.113.217455 PMC3668085

[tpj15923-bib-0030] Shoji, T. , Umemoto, N. & Saito, K. (2021) Genetic divergence in transcriptional regulators of defense metabolism: insight into plant domestication and improvement. Plant Molecular Biology, 109, 401–411. 34114167 10.1007/s11103-021-01159-3

[tpj15923-bib-0031] Shoji, T. & Yuan, L. (2021) ERF gene clusters: working together to regulate metabolism. Trends in Plant Science, 26, 23–32. 32883605 10.1016/j.tplants.2020.07.015

[tpj15923-bib-0032] Sisson, V.A. & Saunders, J.A. (1982) Alkaloid composition of USDA tobacco (*Nicotiana tabacum* L.) introduction collection. Tobacco Science, 26, 117–120.

[tpj15923-bib-0033] Sui, X. , Xie, H. , Tong, Z. , Zhang, H. , Song, Z. , Gao, Y. et al. (2020) Unravel the mystery of *NIC1*‐locus on nicotine biosynthesis regulation in tobacco. bioRxiv. 10.1101/2020.0704.187922

[tpj15923-bib-0034] Valleau, W.D. (1949) Breeding low‐nicotine tobacco. Journal of Agricultural Research, 78, 171–181. 18113660

[tpj15923-bib-0035] WHO . (2015) Advisory note: global nicotine reduction strategy. WHO study group on tobacco product regulation. Geneva: WHO Press.

[tpj15923-bib-0036] Wurtzel, E.T. & Kutchan, T.M. (2016) Plant metabolism, the diverse chemistry set of the future. Science, 353, 1232–1236. 27634523 10.1126/science.aad2062

