# Supplementary material for: Natural and induced variations in transcriptional regulator genes result in low‐nicotine phenotypes in tobacco
Source: Plant J. 2022 Aug 11;111(6):1768–79. doi: 10.1111/tpj.15923 (PMC9544004; doi:10.1111/tpj.15923)
Supplement: Supplementary file 1 — Figure S1. Biosynthetic pathway of nicotine alkaloids in tobacco. Figure S2. Leaf nicotine contents in tobacco lines from the US Nicotiana Germplasm Collection (Sisson & Saunders, 1982). Figure S3. Lines TI1246 and TI1573 have nic1‐1 and nic2‐1 alleles. Figure S4. Deletions of genomic regions including ERF199 in the low‐nicotine lines. Figure S5. Genotyping of nic1‐2 and nic2‐2 alleles generated by CRISPR/Cas9‐mediated editing. Figure S6. Alkaloid contents and expression levels of nicotine biosynthesis genes in 7‐week‐old tobacco plants with the nic1‐4 genotype. Figure S7. Expression levels of ERF189 and ERF199 in the roots and other organs from Nicotiana tabacum TN90. [file TPJ-111-1768-s002.pdf]

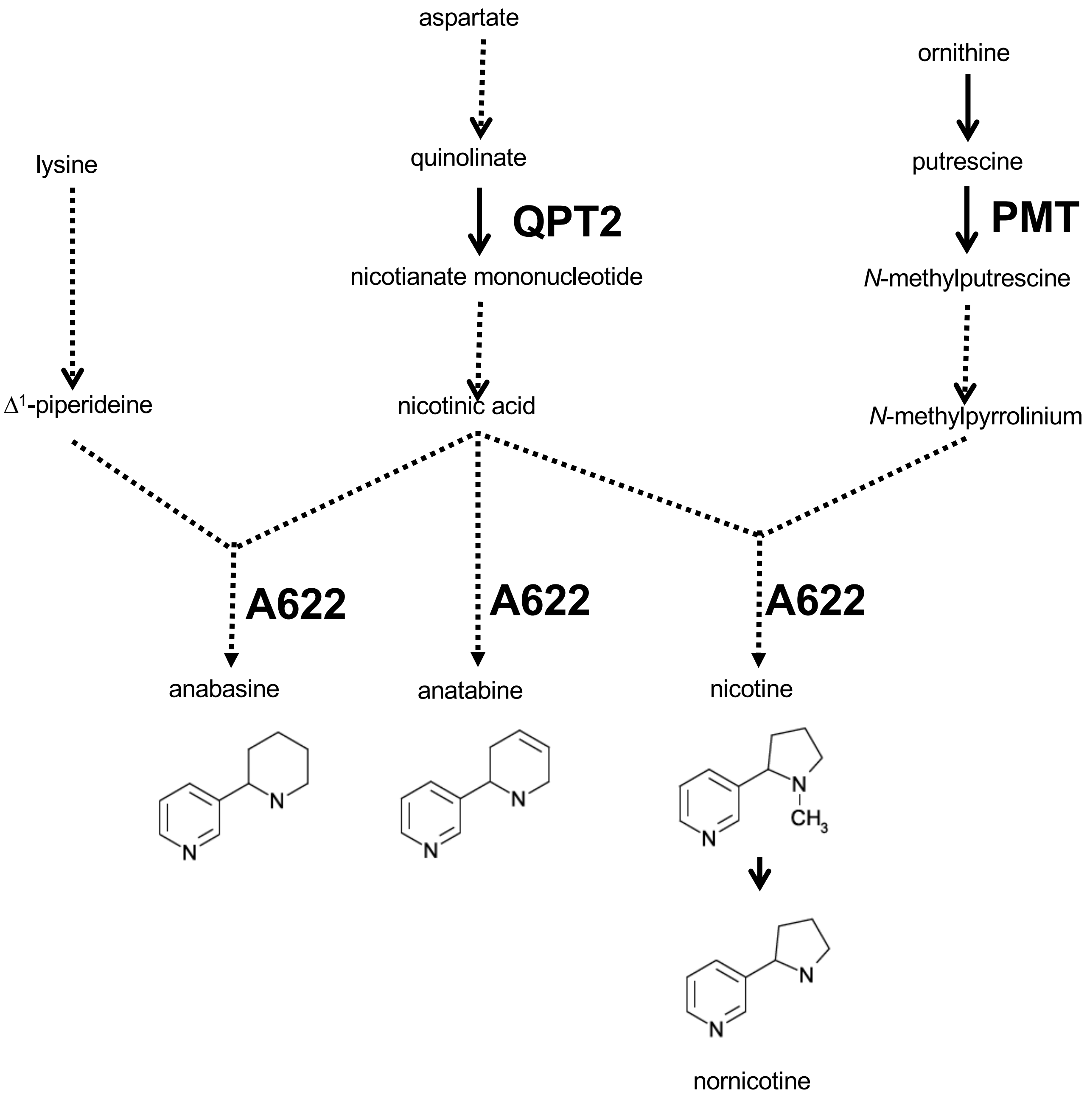

Figure S1 Biosynthetic pathway of nicotine alkaloids in tobacco. Solid arrows indicate defined reaction steps, and dashed arrows indicate undefined or multi-reaction steps [8]. The PIP-family oxidoreductase A622 catalyzes a late step in the pathway, which is required for the production of nicotine, nornicotine, anabasine, and anatabine but has not been defined biochemically [10]. PMT, putrescine *N*-methyltransferase; QPT, quinolinate phosphoribosyl transferase.

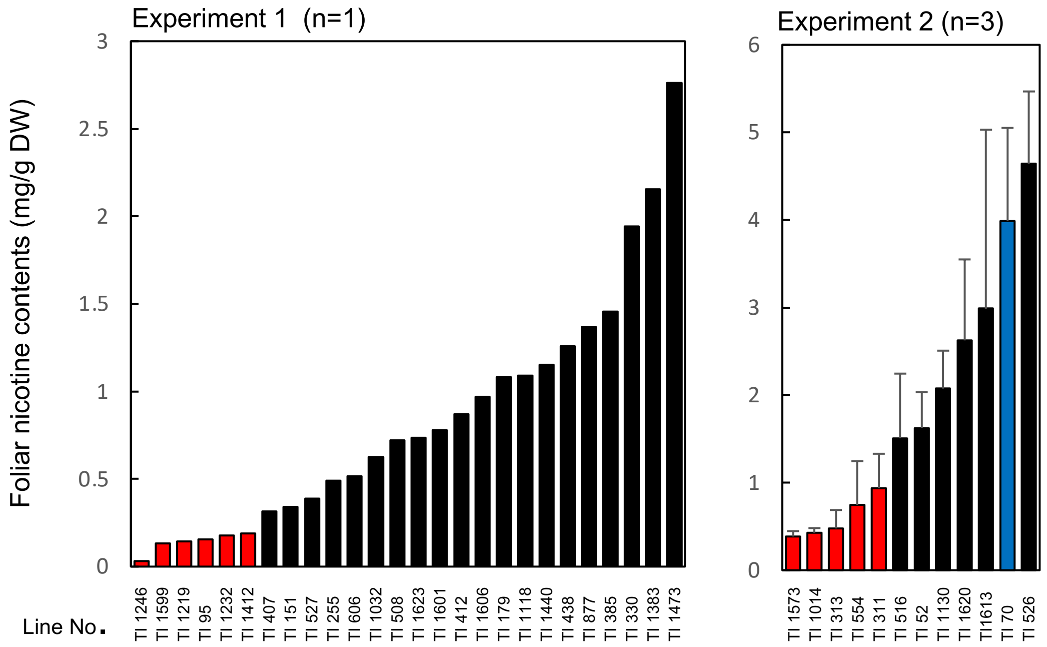

Figure S2 Leaf nicotine contents in tobacco lines from the US *Nicotiana* Germplasm Collection [22]. Ten-week-old plants were examined in the independently conducted Experiments 1 and 2. The average and SD values of three biological replicates are shown for Experiment 2. Line TI70 (blue bar), whose nicotine levels are around the middle of the reported spectrum, was included as a reference. The low-nicotine lines represented by the red bars were studied further.

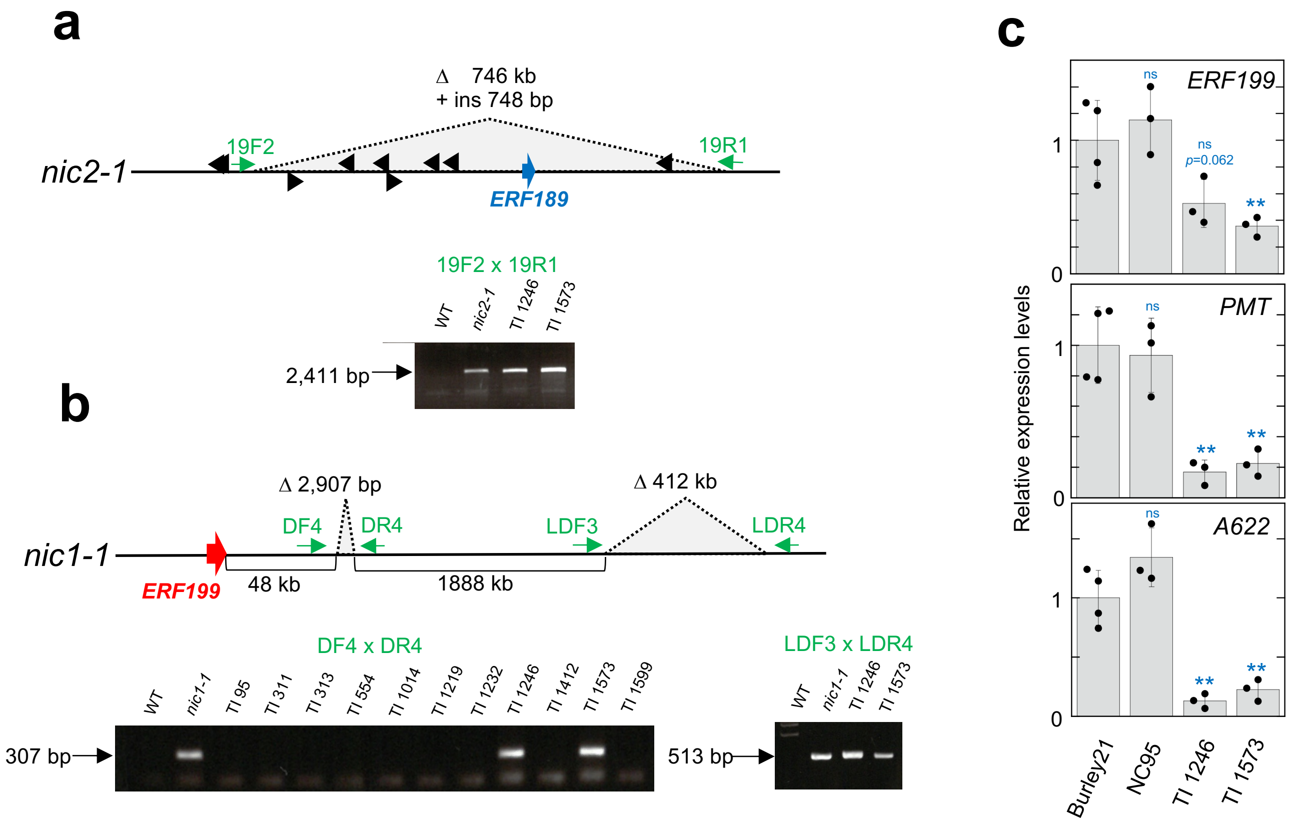

Figure S3 Lines TI1246 and TI1573 have *nic1-1* and *nic2-1* alleles. Schematic diagrams of the deletions in the *nic2-1* (**a**) and *nic1-1* (**b**) alleles. Green arrows indicate the primers used for PCR analysis, the red arrow indicates *ERF199*, the blue arrow indicates *ERF189*, and black arrowheads indicate other *ERF*s clustered in the *NIC2* locus. The deletions were detected by the PCR amplification of genomic fragments (2,411-bp fragment for *nic2-1* and 307- and 513-bp fragments for *nic1-1*) with the indicated primers, while the wild-type intact allele cannot be amplified. Tobacco introduction (TI) lines with low-nicotine contents (**Fig. S2**) and wild-type (WT), *nic1-1*, and *nic2-1* genotypes in the Burley21 background were analyzed. (**c**) Relative expression levels of *ERF199*, *PMT*, and *A622* genes in the TI1246 and TI1573 lines with the *nic1-1 nic2-1* genotype, as determined by RT-qPCR. Wild-type Burley21 and NC95 were used as references. Error bars indicate standard deviation (SD) of the biological replicates. Expression levels in the Burley21 controls were set to 1. Significant differences relative to the controls were determined by Student's *t*-tests; $*P < 0.05$, $**P < 0.01$. *P*-values are given when the differences are not significant (ns).

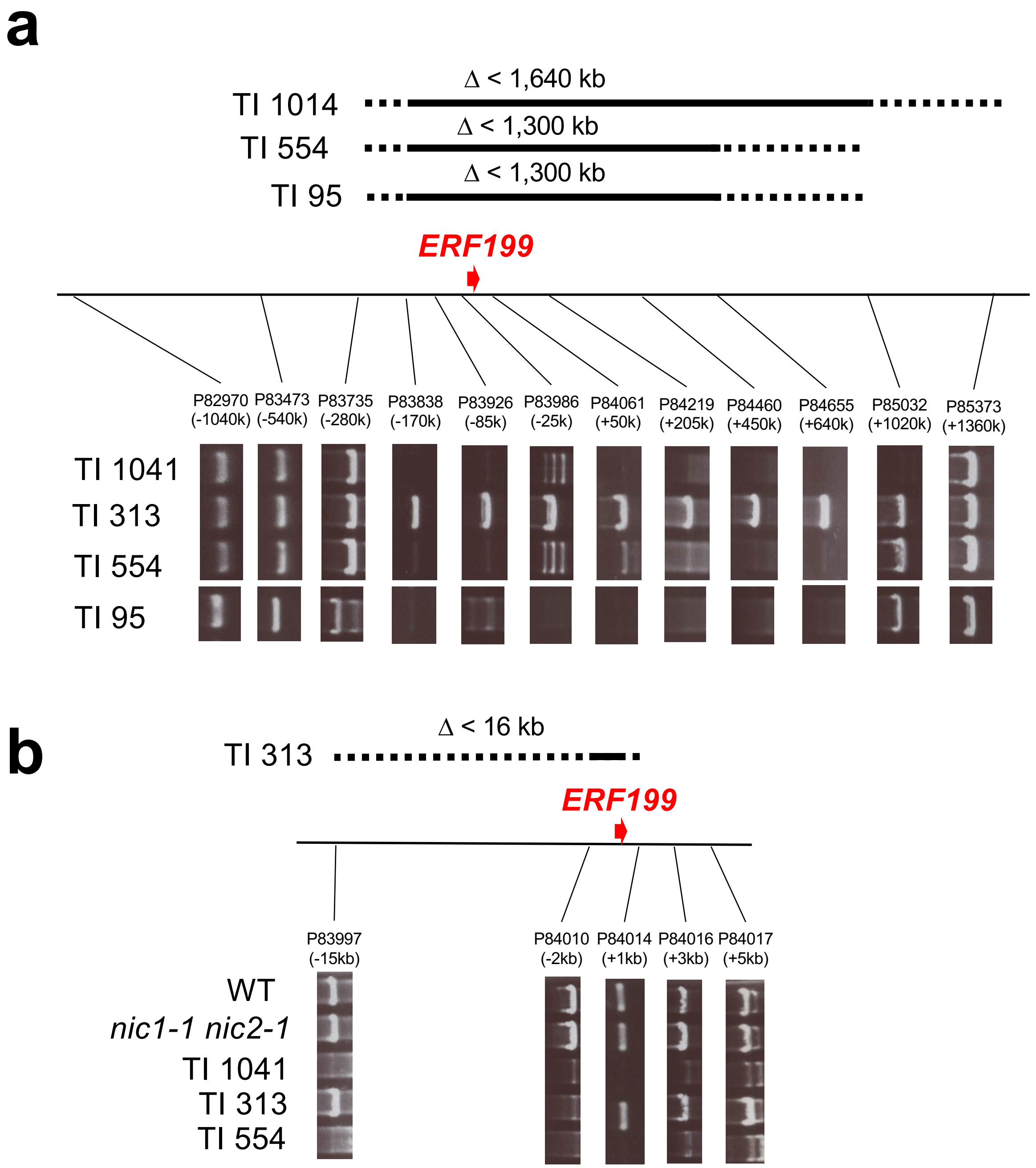

Figure S4 Deletions of genomic regions including *ERF199* in the low-nicotine lines. (**a**) Genomic PCR analysis using 12 pairs of primers targeting the indicated regions around the *NIC1* locus. Schematic diagram of the genomic regions that are possibly deleted. (**b**) Genomic PCR analysis using four pairs of primers targeting the indicated regions around *ERF199*. Schematic diagram of the genomic region possibly deleted in TI313.

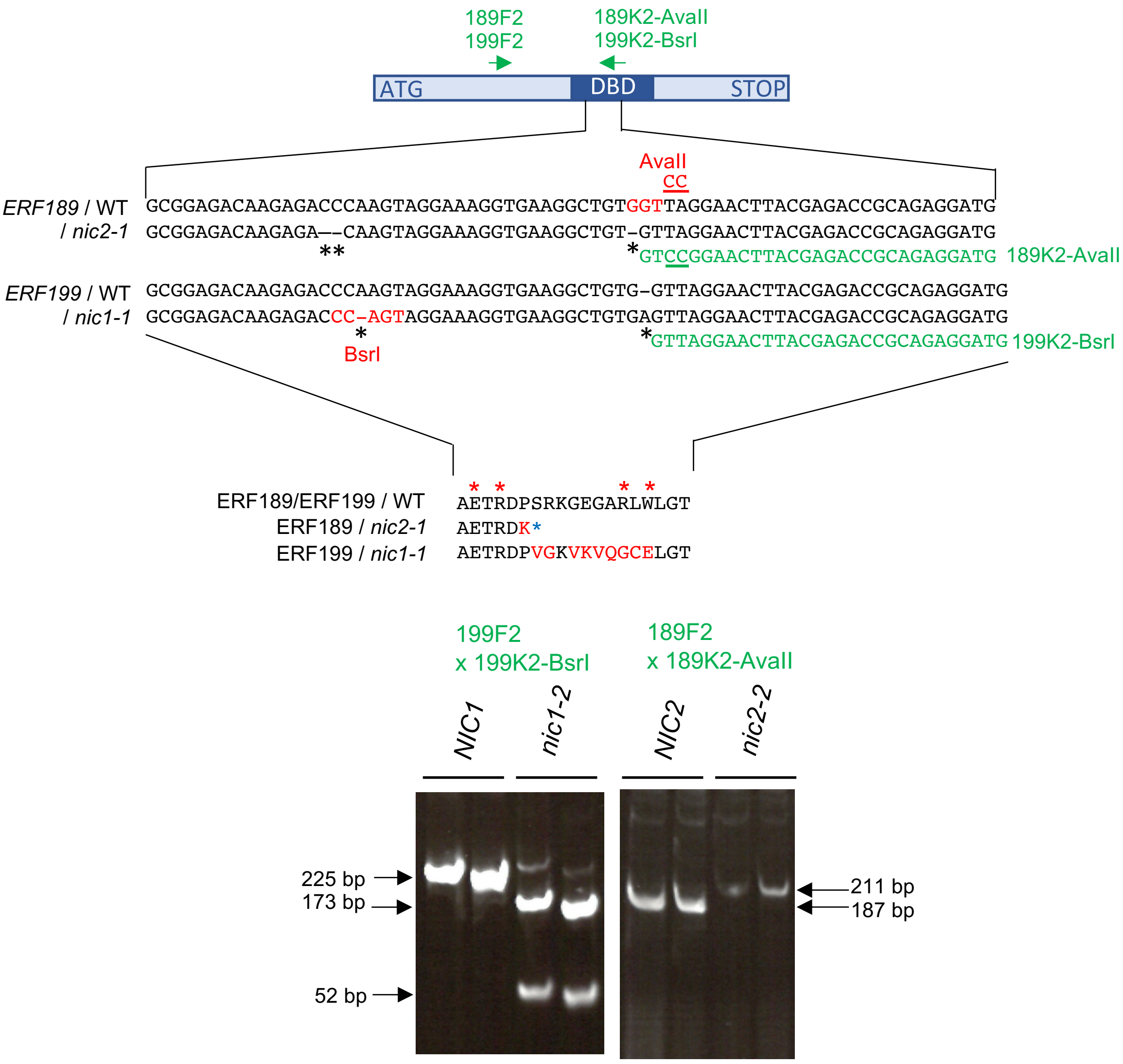

Figure S5 Genotyping of *nic1-2* and *nic2-2* alleles generated by CRISPR/Cas9-mediated editing. Schematic diagrams of the mutations in *ERF189* and *ERF199*. Partial nucleotide and deduced amino acid sequences of *ERF189* and *ERF199* in the wild-type (WT) and the *erf189 erf199* knockout line K2, where the mutations are present, are indicated with the primer sequences (green). The nucleotide positions changed in the mutants (deletions and additions) are denoted with asterisks, while the nucleotides changed with the PCR primer (189K2-*Ava*II) are underlined. Amino acid residues altered in the mutants were shown in red. A stop codon introduced in *nic2-1* and positions of amino acid residues presumed to contact base moiety of DNA (Shoji *et al.* 2013) are shown with blue and red asterisks, respectively. The targeted restriction sites are shown in red. Images of the separated DNA fragments are shown with the expected sizes.

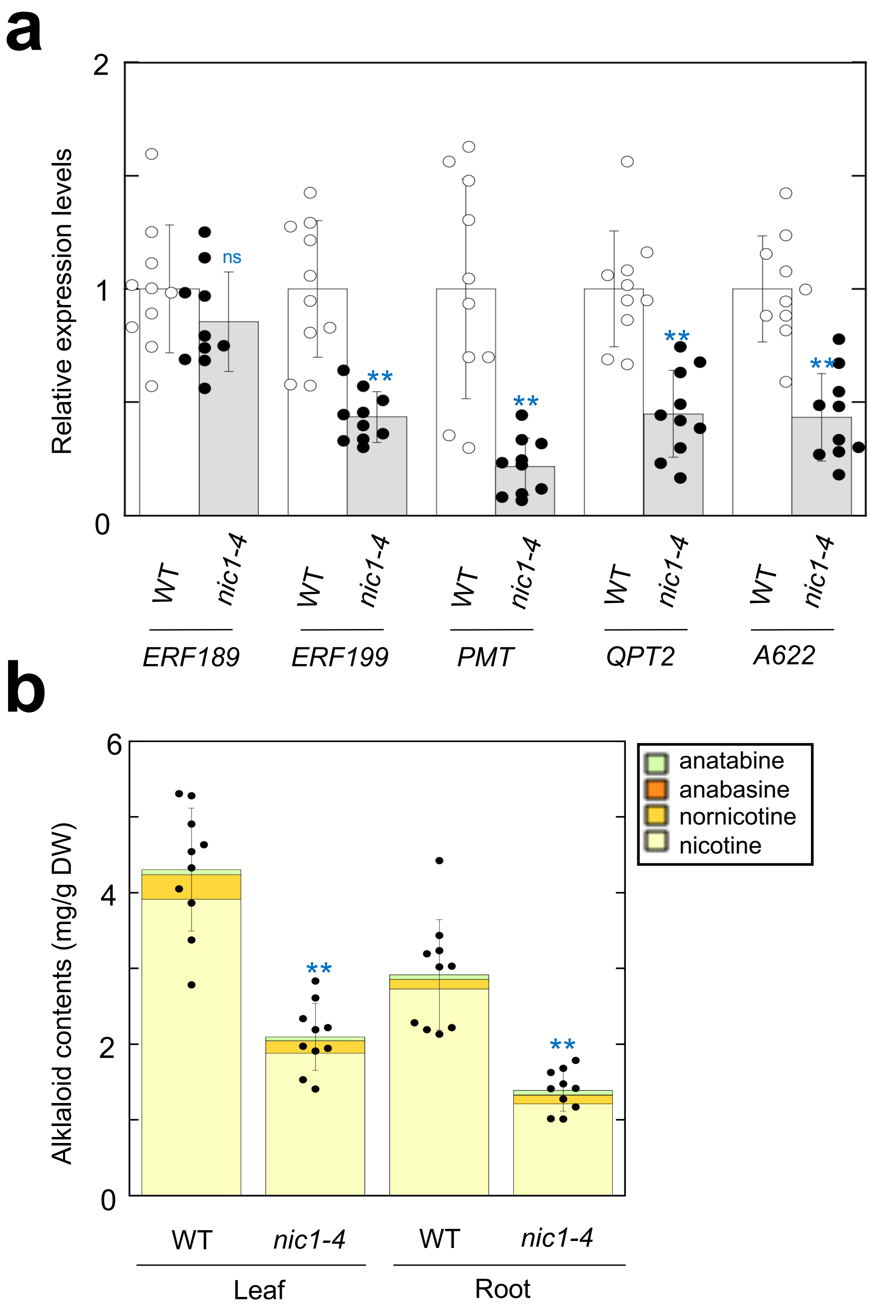

Figure S6 Alkaloid contents and expression levels of nicotine biosynthesis genes in 7-week-old tobacco plants with the *nic1-4* genotype. (**a**) Relative transcript levels in the roots, as analyzed by RT-qPCR. Error bars indicate the SD of the biological replicates. Expression levels in the wild-type (WT) controls were set to 1. (**b**) Alkaloid contents in leaves and roots. Black dots represent total alkaloid contents of individual biological replicates, while error bars indicate their SD. Significant differences relative to the controls were determined by Student's *t*-tests; $*P < 0.05$, $**P < 0.01$. *P*-values are indicated when the differences are not significant (ns).

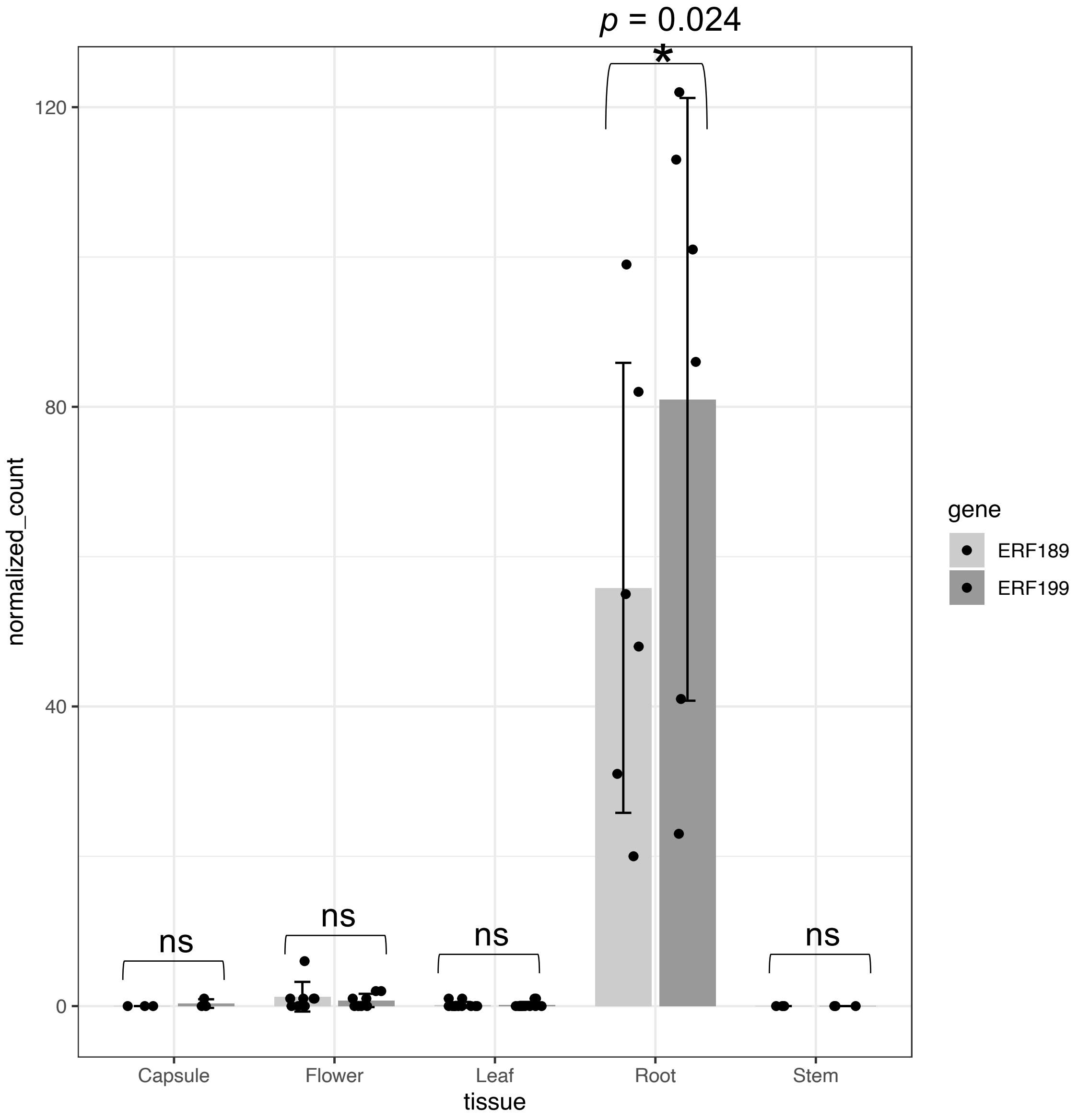

Figure S7 Expression levels of *ERF189* and *ERF199* in the roots and other organs from *Nicotiana tabacum* TN90. Publicly available transcriptome datasets were used for the meta-analysis. Error bars indicate the SD of values from different datasets. Significant differences between the genes were determined by Student's *t*-tests; $*P < 0.05$, $**P < 0.01$. ns, not significant.
