# Supplementary material for: Natural and induced variations in transcriptional regulator genes result in low‐nicotine phenotypes in tobacco
Source: Plant J. 2022 Aug 11;111(6):1768–79. doi: 10.1111/tpj.15923 (PMC9544004; doi:10.1111/tpj.15923)
Supplement: Supplementary file 2 — Table S1. Primers used in genomic PCR analyses. Table S2. Primers used in RT‐qPCR analyses. Table S3. Primers used in bisulfite sequencing. [file TPJ-111-1768-s001.pdf]

Table S1 Primers used in genomic PCR analyses

targets	primer names	sequences (5' to 3')
ERF189	189F	GGGCAATGGAAATGAATCTAGC
	189R	CTTCCTTCCTTTCACATAG
ERF199	199F	CCATTCATTTTCATCCAAACCC
	199R	CGGAGTACTTTTCATGGGATTC
nic2-1	19F2	CATGACTTTTTGACTTGGTCTTTGC
	19R1	CCCTTCTGGTGTGATCGCACCTATAAGG
nic1-1	DF4	ATCTCACAATTATTGGTATTGG
	DR4	GAAATCGCGGAATTTCGAACTCG
	LDF3	CGGAATCGTCGAACGCACCGCTGATCG
	LDR4	TAAGATTTACACACATAGGGTTGGGTCAC
nic1-3	F4	AAGATGATTGCAATTTTAGACAGTG
	R4	CATTTCTGAATCATCATTGAATGGAGC
nic1-4	DF1-3	ATATATACTGGAGATCATTGGTTATCG
	DR2-1	AAACATCATACAGACTCGCTCGTGC
	DF6-1	TAAATATACAAAAGGCCGAATCCG
nic1-2	199F2	CTGATCAGAATCCTTCACATGAATCCC
	199K2-*Bsr* I	CATCCTCTGCGGTCTCGTAAGTTCCTAAC
nic2-2	189F2	GATCAAAATCCTTCACATGAG
	189K2-*Ava* II	CATCCTCTGCGGTCTCGTAAGTTCCGGAC
NIC1	p82970 F	GAGTCATATGCATTGAGAATCTG
	p82970 R	CGAGTTCGCTACTGTAGATGG
	p83473 F	CCTTGCCATTGCTCTACAATTC
	p83473 R	GGTTAGTCATACTTGCAAGCTGC
	p83735 F	CCAAATCAACGTGACAAATGGTGCAGCCAAG
	p83735 R	GGAATGCACGTTTCATATCTCTGTTTCTCCC
	p83926 F	GACACCACTTACCGCTACCTCAAACACCG
	p83926 R	CGCAGGACCATCTTCGTGATCGTGAAGCAC
	p83986 F	GCCATACCAAAACTGGGCACCTCGTGCCTC
	p83986 R	CGACTCCAGAAGATTATGCAAACACAATACCG
	p84061 F	CGTTTGTTGGCACGACATTGGTTGTCTTCGC
	p84061 R	GGAAGCCTGCTTGGTGTTCTGACTTGTGC
	p84219 F	CCACCAATTCGCTCGAATCTGGCCACAAG
	p84219 R	GTGGTGAAAGTGGTGAGCCGAAGGGATTCTG
	p84460 F	CTGCCTGTAATCAATGCACATCCTCGTCG
	p84460 R	CGTCAACAACCTAACAAACACATGCCATGAC
	p84655 F	CGAGGTTCGACTGATTTTGTTCCCAAGGGTG
	p84655 R	GTCACTGTCTCACAAATTGCACACACCGTG
	p85032 F	CACTTGTGACTCTACCATGAGGCAGGGTAC
	p85032 R	GCTATTGTTCCCTTGCCAGGATTTTATTGTG
	p85374 F	CAGAGTTGCAGTTCAAGGTTACATTACATGC
	p85374 R	GCCTTGTGCCACTGACATTAGTTGTGCGAG
	p83997 F	CTCTACGGTCCACAAATTTCTTCTGCGGAC
	p83997 R	CCATTAACCATCTTCATCCCCAAACTCG
	p84010 F	GAGTCGGAGGTATGTCGGACAGTACAGG
	p84010 R	CAGGAAAGTGGAAACCGTGAAGAGCTG
	p84014 F	GCCTTTATCACACCTGTTTGC
	p84014 R	CGAAGCCACATACTTATAAGG
	p84016 F	CTCCTTCAAAATTGACGCACAACGCAGTCG
	p84016 R	GGCAGATTTCGTGGCCGACTTTACGCTGAC
	p84017 F	GTGGACCTATTCACCCACCTAGTGGGTCG
	p84017 R	GTCCAATCTCGACATGACAGGGATCCCATCG

Table S2 Primers used in RT-qPCR analyses

gene names	forward or reverse (F or R)	sequences (5' to 3')
Nt ERF189	F	GCAGCTTCGACTGCAGCTTCCTC
	R	CTCCTCGGACTCGGAGCACTTC
Nt ERF199	F	TTAGCAGCTTCGACTTCGAC
	R	CTCGGAGTACTTTTCATGGG
Nt ERF189/ERF199	F	CCGTGGGGGACGTTTTCGGCGGAG
	R	AACGCTGCATCCTCTGCGGTCTCG
Nt/Nb PMT	F	GAGGAATTGGTTTTACATTATTCG
	R	CTAGATACATCAACTACCACGTC
Nt/Nb QPT2	F	CACAACCAAAGAAAGCATTAAGC
	R	TTTTCACCACCAACCTTGGA
Nt/Nb A622	F	CATAGCGACATACACTATCG
	R	GGCATATGGCCAAATTAGTC
EF1a	F	AAGCCCATGGTTGTTGAGAC
	R	GTCAACGTTCTTGATAACAC

Table S3 Primers used in bisulfite sequencing

amplified regions	primer names	forward or reverse (F or R)	sequences (5' to 3')
-239 to +16	F6	F	AAATAAAAGGTGTAAATATAAAA
	R6	R	TAAAAAACCAATTACRACCCTRCA
-709 to -277	F7	F	TGYAGGGTYGTAATTGGTTTTTA
	R7	R	CCTTATATATTCTCCTCTTTTTT
-239 to +16	F5	F	TGGAGTTAATTTGTATAGTGTTG
	R5	R	CTRAATTCATTTCCATTACC
